# The economic burden of cardiovascular disease and hypertension in low- and middle-income countries: a systematic review

**DOI:** 10.1186/s12889-018-5806-x

**Published:** 2018-08-06

**Authors:** Adrian Gheorghe, Ulla Griffiths, Adrianna Murphy, Helena Legido-Quigley, Peter Lamptey, Pablo Perel

**Affiliations:** 1 Oxford Policy Management Ltd, Level 3 Clarendon House, 52 Cornmarket St, Oxford, OX1 3HJ UK; 2 UNICEF, 3 United Nations Plaza, New York, NY 10017 USA; 3 Centre for Global Chronic Conditions, LSHTM, Keppel Street, London, WC1E 7HT UK; 4 Saw Swee Hock School of Public Health, National University of Singapore, Tahir Foundation Building, 12 Science Drive 2, #10-01, Singapore, 117549 Singapore; 5 Department of Non-communicable Disease Epidemiology, LSHTM, Keppel Street, London, WC1E 7HT UK; 6 Department of Global Health and Development, LSHTM, Keppel Street, London, WC1E 7HT UK; 7 LSHTM, Keppel Street, London, WC1E 7HT UK

**Keywords:** Non-communicable disease, Cardiovascular disease, Hypertension, Economic burden, Systematic review, Low-income, Middle-income

## Abstract

**Background:**

The evidence on the economic burden of cardiovascular disease (CVD) in low- and middle- income countries (LMICs) remains scarce. We conducted a comprehensive systematic review to establish the magnitude and knowledge gaps in relation to the economic burden of CVD and hypertension on households, health systems and the society.

**Methods:**

We included studies using primary or secondary data to produce original economic estimates of the impact of CVD. We searched sixteen electronic databases from 1990 onwards without language restrictions. We appraised the quality of included studies using a seven-question assessment tool.

**Results:**

Eighty-three studies met the inclusion criteria, most of which were single centre retrospective cost studies conducted in secondary care settings. Studies in China, Brazil, India and Mexico contributed together 50% of the total number of economic estimates identified. The quality of the included studies was generally low. Reporting transparency, particularly for cost data sources and results, was poor. The costs per episode for hypertension and generic CVD were fairly homogeneous across studies; ranging between $500 and $1500. In contrast, for coronary heart disease (CHD) and stroke cost estimates were generally higher and more heterogeneous, with several estimates in excess of $5000 per episode. The economic perspective and scope of the study appeared to impact cost estimates for hypertension and generic CVD considerably less than estimates for stroke and CHD. Most studies reported monthly costs for hypertension treatment around $22. Average monthly treatment costs for stroke and CHD ranged between $300 and $1000, however variability across estimates was high. In most LMICs both the annual cost of care and the cost of an acute episode exceed many times the total health expenditure per capita.

**Conclusions:**

The existing evidence on the economic burden of CVD in LMICs does not appear aligned with policy priorities in terms of research volume, pathologies studied and methodological quality. Not only is more economic research needed to fill the existing gaps, but research quality needs to be drastically improved. More broadly, national-level studies with appropriate sample sizes and adequate incorporation of indirect costs need to replace small-scale, institutional, retrospective cost studies.

**Electronic supplementary material:**

The online version of this article (10.1186/s12889-018-5806-x) contains supplementary material, which is available to authorized users.

## Background

Globally, non-communicable diseases (NCDs) account for more than 60% of disability-adjusted life years (DALYs), 70% of deaths and more than 80% of years lived with disability (YLD) [1, 2]. Cardiovascular disease (CVD) represents 24% of NCD-related DALYs, with ischemic heart disease and cerebrovascular disease the two major causes of disability globally. Although age-standardised CVD mortality rates have been declining globally by 14.5% between 2006 and 2016 [3], the burden of CVD remains disproportionately larger in low- and middle-income countries (LMICs) compared to high-income countries (HICs) as more than 80% of CVD deaths occur in LMICs [4–6]. Moreover, CVD affects working age populations much more in LMICs compared to HICs. For example, in Sub-Saharan Africa half of cardiovascular deaths occur in the 30–69 years age group, at least ten years earlier than in HICs [7]. The total economic loss due to CVD in LMICs was estimated to amount to $3.7 trillion (2010) between 2011 and 2015, representing approximately half the NCD economic burden and 2% of Gross Domestic Product (GDP) across LMICs [8].

Despite high-level political commitment to improve cardiovascular disease outcomes by 2025 [9], most countries are off course to meet the targets [10]. In many LMICs, particularly in the poorest settings, even meeting all the globally adopted risk factor targets may not be sufficient to achieve the global target of 25% reduction in CVD mortality by 2025 [11]. The challenges of health systems in LMICs – insufficient health spending, poor governance, inefficient care delivery systems, focus on curative care at the expense of prevention, to name just a few factors – have been suggested to contribute to the CVD burden much more than the risk factor levels, which remain low in LMICs compared to HICs [12]. For example, CVD secondary prevention medicines remain unavailable and unaffordable in many of these countries [13].

Disability caused by CVD has economic consequences at multiple levels: individual, household, economic agents, public institutions, government and the society as a whole. Not only is this burden expected to increase in the future [14], but LMICs will incur an increasing share of this burden due to population growth, ageing and globalization. The economic impact of CVD on households, health systems and national incomes in LMICs may jeopardise the ongoing poverty reduction initiatives [15, 16]. Given the centrality of advocacy efforts towards governments by professionals and patients for improving outcomes in LMICs [12], the importance of good quality economic data is undisputed. However, the available economic modelling studies providing estimates on the economic burden of CVD in LMICs [17, 18] were informed by selective literature reviews and the existing economic data appear to be insufficient and of questionable quality [19].

Our objective was to address this knowledge gap by synthesising the available data on the economic burden of CVD and hypertension in LMICs to households, the health system and society. To this end, we systematically reviewed the existing evidence as part of a larger review of the economic burden of seven major NCD categories in LMICs (cardiovascular disease, diabetes, respiratory disease, cancer, neurological disease, psychiatric disorders and musculoskeletal disorders). The findings of this CVD and hypertension review aim to inform the formulation and refinement of policy and research objectives in this area, as well as to synthesize available data for future economic modelling exercises.

## Methods

The systematic review was conducted according to a pre-specified protocol available from the authors upon request. The review is registered on the PROSPERO register of systematic reviews (ID CRD42014005346). The review is reported according to the PRISMA statement [20].

### Search strategy and selection criteria

We included studies using primary or secondary data to produce original economic estimates of the impact of CVD. For the purpose of this review, we included cardiovascular disorders together with hypertension as the leading CVD risk factor [21] – other CVD risk factors (e.g. smoking, diabetes, cholesterol) were excluded. Specifically, we included observational designs with a cost collection component, cost-of-illness studies, and economic modelling studies. Studies reporting any microeconomic outcome expressed in monetary units (e.g. cost of disease management per patient year, nationwide costs of disease management per year) or macroeconomic outcome (e.g. cost of disease as %GDP) were included. The only non-monetary microeconomic outcome accepted was productive time lost due to illness (e.g. number of days off work). Multi-country studies referring to a mix of LMICs and HICs were included only subject to providing explicit and detailed economic estimates for the considered LMICs. The following categories of sources were excluded: literature reviews, policy papers, editorials, commentaries, opinion pieces, and economic evaluation studies.

We searched 16 electronic databases from 1990 onwards: MEDLINE, MEDLINE In-Process and Other Non-Indexed Citations (Ovid); EMBASE; NHS Economic Evaluation Database (NHSEED); EconLit; Cochrane Central Register of Controlled Trials (CENTRAL); Cochrane Database of Abstracts of Reviews of Effects (DARE); PsycInfo (Ovid); Latin American and Caribbean Health Sciences Literature database (LILACS); MedCarib; Africa-Wide Information; Global Health; Index Medicus for the South-East Region (IMSER); Index Medicus for the Eastern Mediterranean Region (IMSEAR); Western Pacific Region Index Medicus (WPRIM); and the New York Academy of Medicine Grey Literature Report. In addition, we searched the websites of the World Health Organization, the World Bank, the World Economic Forum, and NCD Alliance for relevant reports. We also hand-searched the reference lists of included studies for further relevant sources. No language restrictions applied. The initial search was conducted in August 2013 and updated in June 2015.

Our search strategy (presented in full in Additional file 1) combined three groups of search terms: 1) CVD and hypertension; 2) LMICs - 139 LMICs were identified based on the World Bank income classification of countries and searched individually, as well as with generic terms such as ‘low-income’ and ‘middle-income’; and 3) economic burden - the following generic search terms were used: burden of illness; cost of illness; health expenditure; costs and cost analysis; absenteeism; productivity loss; poverty; income; economic modelling; economic burden; resource utilization; employment; labour.

Study selection followed a three-stage process: 1) two independent reviewers performed title & abstract screening and excluded irrelevant studies; 2) two independent reviewers screened the full-text articles against the inclusion/exclusion criteria; and 3) the reference lists of included studies were hand-searched to identify any other relevant publications. The remaining studies at the end of Stage 2 and any studies identified in Stage 3 were included in the review. At all stages disagreements between reviewers were discussed and resolved consensually. If necessary, the opinion of a third reviewer was elicited.

### Quality assessment and data extraction

We appraised the quality of included studies using a seven-question quality assessment tool developed for the purpose of this study (Additional file 2). The tool focussed on two aspects: the design of the economic study, inspired by the CHEERS (Consolidated Health Economic Evaluation Reporting Standards) Statement [22]; and, if applicable, the design of the epidemiological study alongside which the economic study was conducted.

We extracted data on 1) methodological characteristics of studies: geographical setting and any relevant contextual information; scope (institutional; regional; national; international); disease area(s); economic perspective (provider/payer/societal/other); type of study design (Table 1); population characteristics; study design characteristics; study duration/time horizon; type(s) of economic estimates reported; and 2) economic estimates, by cost component (direct/indirect – the latter comprising productivity costs and costs associated with complications and comorbidities; medical/non-medical; macroeconomic indicators). A data extraction template was developed and piloted on a randomly selected sample of four included studies. Data were extracted by one researcher and checked by others. Disagreements were resolved by consensus.

**Table 1:** Definitions used to categorise included studies

Study design	
prospective cost study	cost study informed by data gathered from a prospective cohort of patients
retrospective cost study	cost study informed by data collected from a retrospective cohort of patients, e.g. medical records, case notes
database analysis	analysis of patient records from an already existing database, e.g. health insurance claims, hospital reimbursements
mathematical model	mathematical model (+/− simulation) extrapolating primary data to produce original estimates beyond their original scope e.g. time and location
survey	cross-sectional study of patients +/− controls
COI study	cost-of-illness study evaluating the region or country-level economic consequences that the presence of disease and its outcomes exert on individuals and society as a whole
Study scope	
Institutional	Study conducted in one or more health care facilities, with no specified geographical scope below national level and no evidence of a sampling procedure to ensure representativeness
City	Study conducted in health care facilities in a specified city
Regional	Study conducted in health care facilities in a specified sub-national administrative unit (e.g. region, province, state)
National	Study conducted at the national level, either through representative sampling of health care facilities or through modelling
International	Multi-country study
Other	Other than above or multiple categories
Economic perspective	
Patient	Study reporting direct costs incurred only by patients (e.g. out-of-pocket payments)
Provider	Study reporting costs incurred by the health care provider (e.g. average unit cost of an inpatient day)
Third-party payer	Study reporting costs incurred at the level of a third-party payer (e.g. insurance fund, Ministry of Health vertical programme)
Societal	Study reporting some form of indirect cost, incurred at any level (e.g. value of lost productivity due to illness, effect of CVD on national income)
Other	Other perspectives (e.g. pharmaceutical sector) or multiple perspectives (e.g. patient and provider)
Unclear	Could not be determined based on the information provided

All cost data were converted to purchasing power parity (PPP)-adjusted US$ 2014 (Int$ 2014) using the Campbell and Cochrane Economics Methods Group Evidence for Policy and Practice Information and Coordination Centre (CCEMG-EPPI Centre) cost converter [23]. When the base year of the currency was not reported or could not be inferred from the manuscript (e.g. last year of data collection), it was assumed to be the year before the publication of the paper. For studies presenting estimates for more than one year, the most recent estimate was extracted. No adjustments were made to economic outcomes expressed as ratios or percentages e.g. % of GDP.

### Analysis

We aggregated and synthesized information on general study characteristics (geographical setting, care delivery setting, and pathology), methodological characteristics (study design, population characteristics, control group, sample size, dealing with heterogeneity) and economic burden estimates (currency and year, cost component, cost perspective). We conducted a narrative synthesis focusing on the overall economic burden of CVD and hypertension on households, health systems and the society, as well as current research gaps. Data were managed and analysed using Microsoft Excel and R 3.1 statistical software [24].

### Role of the funding source

There was no funding source for this study. The corresponding author had full access to all the data in the study and had final responsibility for the decision to submit for publication.

## Results

**Fig. 1:**
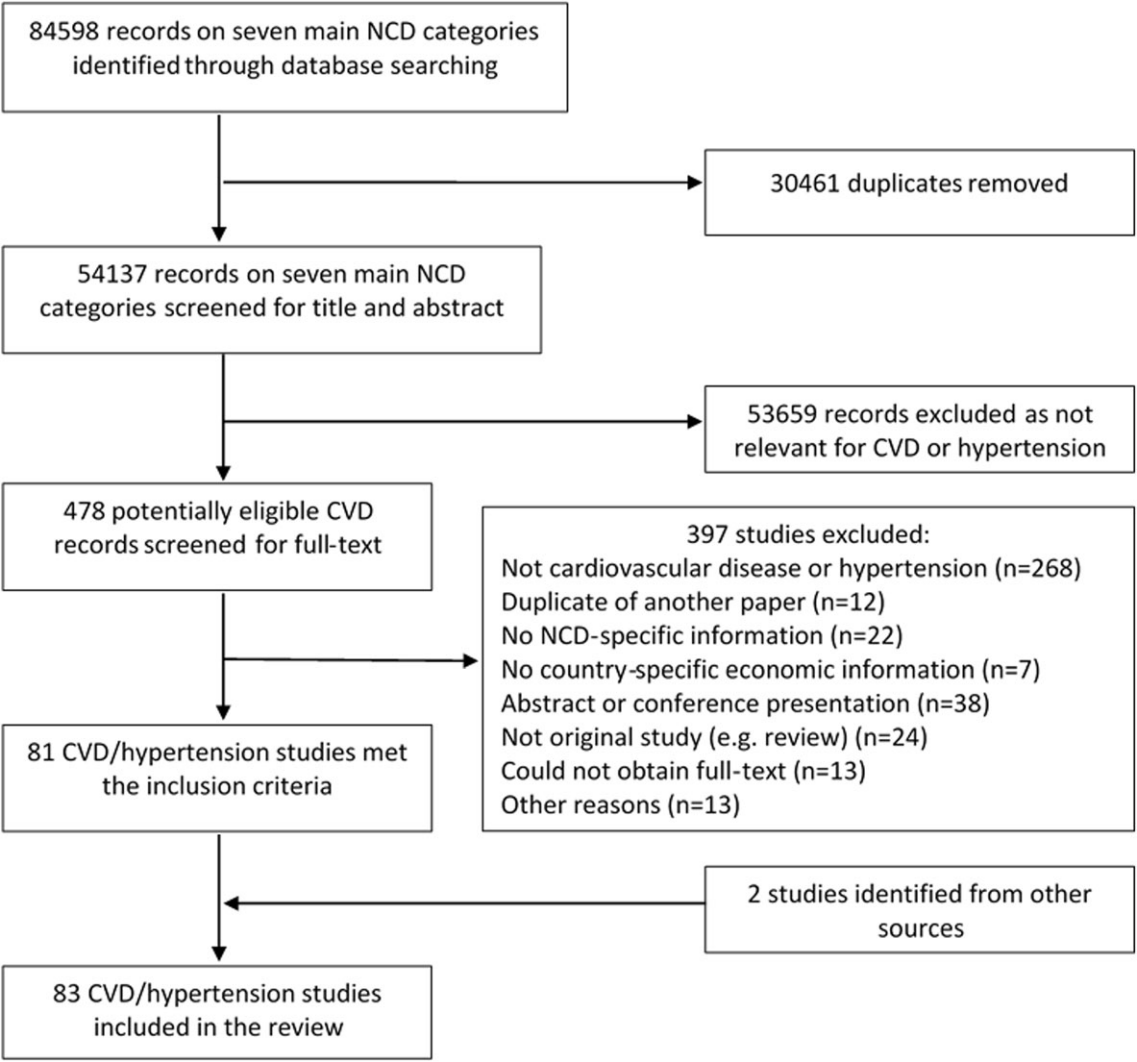
PRISMA flowchart

The initial search across all seven NCDs returned 64,952 records and 19,646 further records were identified at the June 2015 update, leading to a total of 84,598 titles. After excluding the duplicates, 54,137 titles and abstracts were screened for eligibility and 478 studies potentially relevant for CVD and hypertension were screened for full-text. 81 studies met the inclusion criteria and two further studies were identified through manual searches, leading to a total of 83 included papers, reporting economic estimates for/from 28 countries (Fig. 1). Details on the included studies together with data extracted from each full-text source are presented in Additional file 3 and Additional file 4.

### Study characteristics

Key study characteristics are summarised in Table 2. Most studies examined the economic impact of stroke (29%, *n* = 24) and 20% of studies looked at generic CVD, i.e. either unspecified cardiovascular condition(s) or a multitude of cardiovascular disorders for which economic estimates were reported without disaggregation by pathology. Secondary care was the most common study setting: 46% of studies (*n* = 38) were conducted in secondary and outpatient care jointly, while 26% (*n* = 22) looked at secondary care only and 7% (*n* = 6) focused on primary care alone. Five studies included an explicit non-CVD control group, while the others did not have a control group.

**Table 2:** Summary characteristics of included studies (*n* = 83)

Characteristic/level	No. studies (%)
Study scope	
Institutional	40 (48)
National	30 (36)
Regional	9 (11)
City	1 (1)
Other	1 (1)
International	2 (1)
CVD category	
Stroke	24 (29)
Hypertension	18 (22)
Coronary heart disease	17 (20)
Generic CVD	20 (24)
Heart failure	4 (5)
Care setting	
Secondary + outpatient	38 (46)
Secondary	22 (26)
Tertiary	17 (20)
Primary	6 (7)
Study design	
Retrospective cost study	34 (43)
Cost-of-illness study	14 (18)
Database analysis	11 (14)
Prospective cost study	10 (13)
Mathematical model	7 (9)
Other	4 (5)
Included a control group [YES]	5 (6)
Economic perspective	
Provider	29 (35)
Societal	18 (22)
Patient	17 (21)
Other	10 (12)
Third-party payer	7 (9)
Unclear	1 (1)

Most studies were institutional (48%, *n* = 40), i.e. they were conducted in either one health facility (40%, *n* = 33) or a limited number of facilities (8%, *n* = 7). The remaining were nationwide studies (36%, *n* = 30), within-country regional (11%, *n* = 9) or others e.g. city or international. Retrospective cost studies dominated the sample (43%, *n* = 34) to the detriment of cost-of-illness studies (18%, *n* = 14), database analyses (14%, *n* = 11) or prospective cost studies (13%, *n* = 10). The majority of included papers were published after 2005 (76%, *n* = 63). Of the 28 countries for which at least one economic estimate was available, the most estimates were reported for China (20%, *n* = 17), India (12%, *n* = 10), Brazil (10%, *n* = 8) and Mexico (10%, n = 8).

### Quality assessment

Table 3 summarizes the quality assessment. Most studies did not use a sampling method conducive to generalizable results. In most cases, the quality of the CVD incidence/prevalence data source (i.e. how CVD was/had been diagnosed in study patients) could not be assessed. Two thirds of the studies included an exploration of uncertainty and/or heterogeneity in the economic estimates. This often took the form of regression analysis. Data sources for expenditure, resource use and unit costs were clearly presented in 57% (*n* = 47) of studies. Productivity costs were included in estimation and cost data were transparently presented in less than a third of the included papers. Most studies (*n* = 29, 35%) reported costs from the health care provider’s perspective. A patient and a societal perspective were adopted in 21% (*n* = 17) and 22% (*n* = 18) of studies, respectively. The majority of studies reported direct costs only. However, 23 studies also reported estimates of indirect costs. One study reported the effects of CVD on absenteeism, e.g. number of productive days lost due to illness, but did not attempt to calculate indirect costs associated with lost days.

**Table 3:** Summary quality assessment of included studies

Quality criterion	Studies (n, %)		
	Yes	No	Unclear
Economic component			
Data sources for expenditure, resource use and unit costs were clearly explained	48 (58%)	34 (42%)	n/a
Cost and/or productivity data were transparently presented	24 (29%)	59 (71%)	n/a
Productivity costs were included	23 (28%)	60 (72%)	0 (0%)
If productivity costs included, results were presented with and without productivity costs	20 (24%)	3 (4%)	n/a
The analysis addressed uncertainty and/or heterogeneity	53 (64%)	30 (36%)	0 (0%)
Epidemiologic component			
The source of incidence/prevalence data contributed to the study’s internal validity	35 (42%)	13 (16%)	28 (34%)
The patient sampling method appropriate for deriving nationwide estimates of incidence/prevalence	21 (25%)	49 (58%)	9 (11%)

Total number of studies does not add to *n* = 83 in all criteria because certain criteria were not applicable in several studies. Please see the Additional file containing the extraction sheet. n/a - not applicable

### Economic findings

**Fig. 2:**
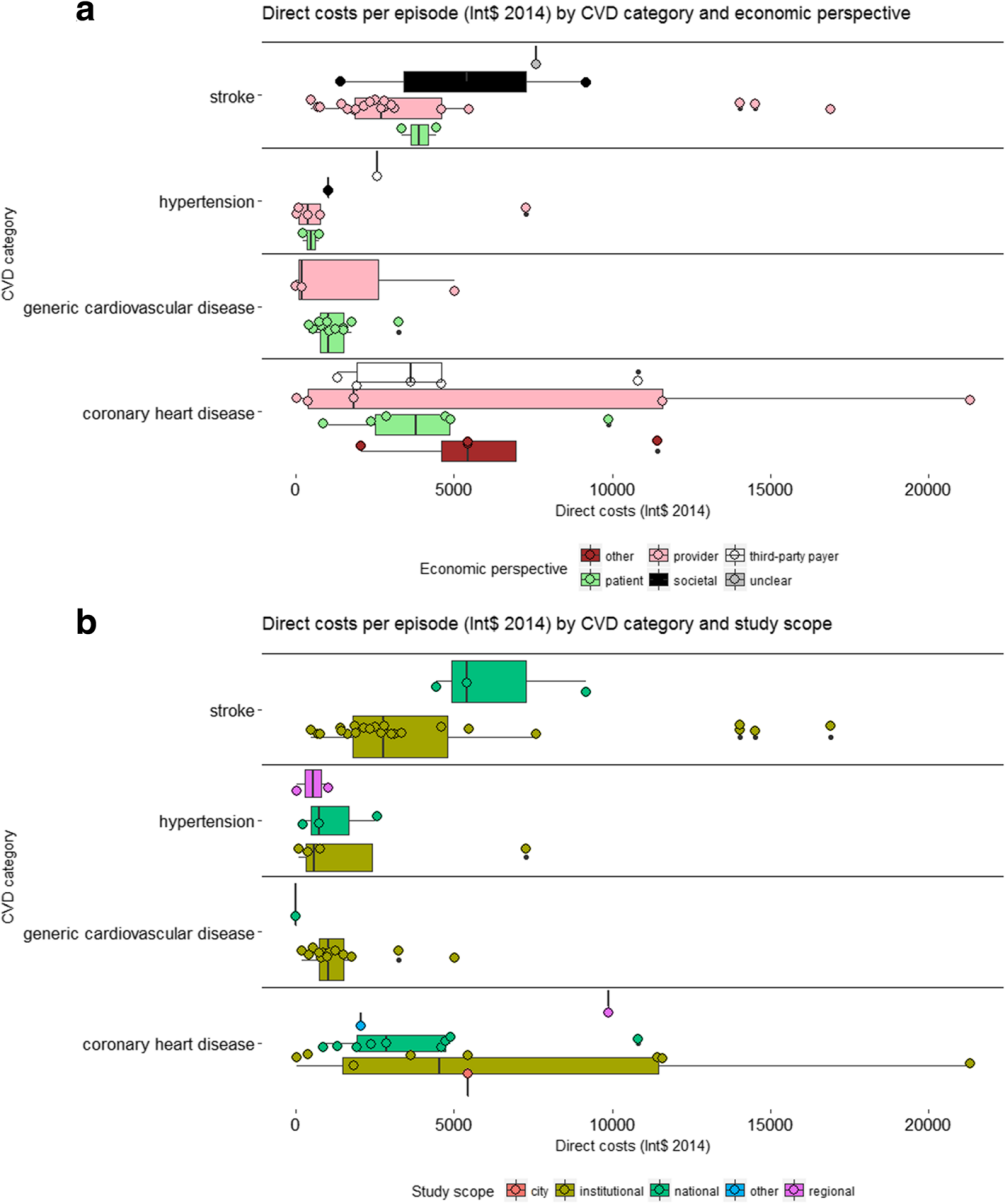
Direct medical costs (Int$ 2014) per episode by CVD category (*n* = 42 studies). 1) For each CVD category and economic perspective (Panel **a**) or study scope (Panel **b**), the boxplot represents cost estimates from individual studies (circles), the sub-group median (vertical line in solid box), inter-quartile range IQR (distance between lower and upper hinges, which correspond to the first and third quartile) and the upper (lower) whisker extends from the hinge to the largest (lowest) observed value no further than 1.5 * IQR from the hinge; 2) Three cost estimates ranging between Int$42,000 and Int$67,000, for very complex interventions in coronary acute syndrome patients (Moleregpoom et al., 2007; Thailand), were excluded from this Figure for ease of visualization. The median cost estimated in the same study for the simplest coronary acute syndrome cases is represented

We distinguish between economic estimates that refer to a distinct episode of care and those referring to the total cost over a specified period, e.g. one month or one year. In Fig. 2 we present the direct costs (Int$ 2014) per CVD episode by cardiovascular pathology, economic perspective (Panel A) and study scope (Panel B). ‘Episode’ refers here to costs associated with any finite interaction with a healthcare provider analysed in the included studies, e.g. consultation, clinic visit or inpatient stay, usually for acute episodes. The costs per episode for hypertension and generic CVD were generally homogeneous across studies, ranging between $500 and $1500. In contrast, for coronary heart disease (CHD) and stroke, cost estimates were higher and more heterogeneous, with several estimates in excess of $5000 per episode. The economic perspective and scope of the study appeared to impact cost estimates for hypertension and generic CVD much less than estimates for stroke and CHD. For example, most institutional-level stroke studies tended to underestimate direct costs per episode relative to national-level studies. Studies on stroke and CHD conducted from the perspective of the patient tended to estimate higher direct costs than studies taking the perspective of the provider.

**Fig. 3:**
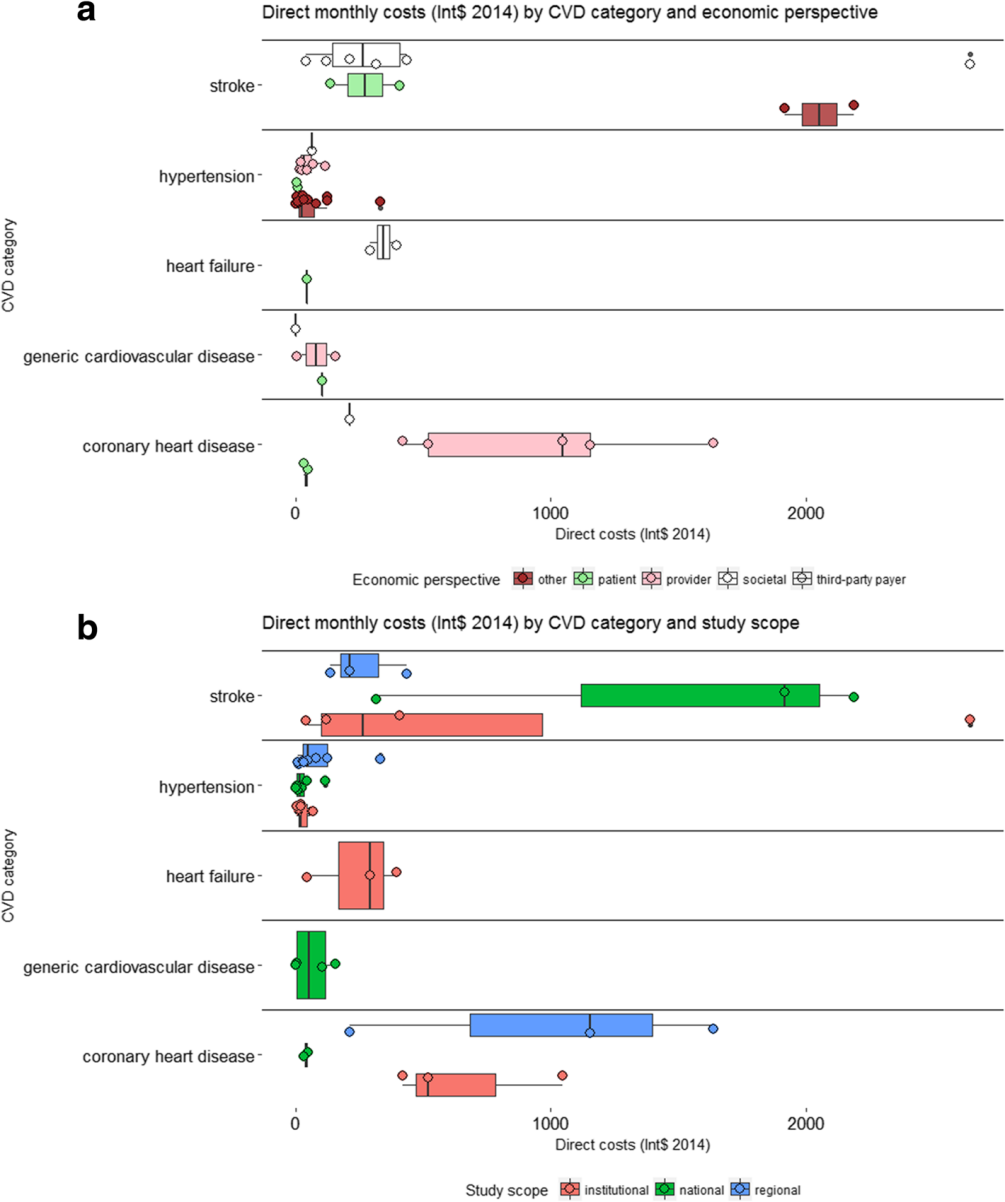
Monthly direct medical costs (Int$ 2014) by CVD category (*n* = 31 studies). For each CVD category and economic perspective (Panel **a**) or study scope (Panel **b**), the boxplot represents cost estimates from individual studies (circles), the sub-group median (vertical line in solid box), inter-quartile range IQR (distance between lower and upper hinges, which correspond to the first and third quartile) and the upper (lower) whisker extends from the hinge to the largest (lowest) observed value no further than 1.5 * IQR from the hinge

We present direct monthly costs for long-term care in Fig. 3. For studies which reported costs for time horizons longer than one month (e.g. six months or one year), we estimated the monthly cost by dividing the total cost by the respective number of months, e.g. annual costs were divided by 12. Most studies reported costs for hypertension, for a median of $22 per month across estimates. The medians for average monthly treatment costs for stroke and CHD were higher, but varied with study scope and economic perspective from as little as $50 per month (e.g. CHD, patient perspective) to over $1000 (e.g. CHD, provider perspective). However the number of data points for CHD, stroke and heart failure was much more limited than for hypertension and variability across estimates was higher.

Table 4 attempts to synthesize the magnitude of the economic burden for different CVD categories and hypertension, based on the disease categories reported by the study authors. Studies where a detailed categorization was not reported, or which grouped several CVD categories together without reporting separate estimates for each, were grouped under “generic CVD”. For each country-specific estimate of the total cost per episode or total annual cost of care, we calculated the ratio total cost: total health expenditure per capita (Int$). For example, the annual direct cost per patient of hypertension could be as high as 5.9 times the total health expenditure per capita, with a median ratio of 0.7. The higher the ratio, the higher the economic burden of disease. Country-specific ratios are presented in Additional file 5.

**Table 4:** Ratio of total cost of care to country-specific total health expenditure per capita (Int$), summary statistics

NCD category	Min	Median	Max	No studies
Annual cost of care				
coronary heart disease	1.46	10.02	27.83	5
generic CVD	0.01	0.96	3.29	4
chronic heart failure	5.59	9.41	56.98	3
hypertension	0.01	0.68	5.89	10
stroke	1.48	12.70	472.48	9
Cost per episode				
coronary heart disease	0.26	12.73	143.38	14
generic CVD	0.17	4.07	47.20	4
hypertension	0.09	3.84	52.63	8
stroke	0.72	10.44	497.06	16

Several studies reported macroeconomic estimates. Specifically, six studies reported the national or regional economic loss due to CVD in sub-Saharan Africa [25] ($9bn), Brazil [26] ($ 20bn), India [27] ($2.4 trillion over 2012–2030), Fiji Islands [28] ($8.5mn), Serbia [29] ($1bn) and China [27] ($8.8trillion over 2012–2030). One paper used econometric methods to estimate the global cost of heart failure to be $15.6 billion, however data from only one LMIC (Brazil) informed the model, the rest being high-income countries [30]. Four studies reported the cost of CVD as a proportion of national or regional GDP: Brazil [26] (1.7%), sub-Saharan Africa [25] (7%), Russian Federation [31] (2.8%) and Serbia [29] (1.8%).

## Discussion

### Summary of findings

We identified a heterogeneous body of literature on the economic burden of CVD and hypertension in LMICs, dominated by single centre retrospective cost studies conducted in secondary care settings. The evidence base has been growing over time, particularly after 2010, and appears to be concentrated in several countries: studies in China, Brazil, India and Mexico combined contributed half the economic results across all included studies.

We identified several gaps in the available literature. First, a geographical gap: there is a dearth of economic estimates from sub-Saharan Africa, Central America and Eastern Europe. This is a concern because the research does not reflect current and upcoming disease burden trends. Furthermore, countries with one or two studies are often informed by data collected in one service provider, which seriously limits their generalisability and usefulness for national decision-makers. Second, there is a gap relating to delivery setting: most studies concentrated on hospital care, with very few studies including economic aspects of CVD care in primary and community care. Only two studies estimated the costs of prevention, one in Mexico [32] and another in China [33]. Given the importance of prevention and service delivery at these levels, particularly as more innovative models are being rolled out at these levels in low-resource settings, there would be great value in exploring them as a matter of priority [9]. Third, a research design gap is also apparent: the majority of the included studies were retrospective cohorts without a control group and did not estimate indirect costs, making it difficult to isolate the economic burden attributable to CVD or hypertension. This is particularly concerning given the long-term consequences of CVD care for patients and their carers. Finally, there is also a research quality gap: the quality of the included studies was generally low. Reporting transparency, particularly for cost data sources and results, was poor.

### Strengths and limitations

While our findings generally agree with those previous reviews [34–36], they expand them in several ways: we go beyond stroke and ischemic heart disease to include hypertension and heart failure; we find that the annual cost of care and the cost of an acute episode for patients with CVD can exceed many times the total health expenditure per capita in LMICs; and we distinguish between the cost of inpatient episodes and the monthly cost of non-acute care. Methodologically, the value of our review lies in the wide-ranging systematic search strategy, the absence of language restrictions, the broad time horizon (1990 onwards), the range of economic perspectives considered (from household to societal), the attempt to synthesize comparatively the economic impact of different pathologies, as well as the quality assessment of included studies. None of the previous reviews shares all these attributes. We believe our results provide the first comprehensive picture of the breadth and quality of economic research into CVD and hypertension in LMICs.

There are also limitations. First, although we devised as sensitive a search strategy as possible, we acknowledge there may be grey literature sources that we did not identify. In the same vein, we did not exclude studies on quality considerations, which may have led to the overall quality of included studies to be lower than warranted. Nevertheless, we believe there is value in synthesizing information over the entire body of research at this stage in order to inform future studies. Second, we excluded economic evaluation studies for two types of reasons: methodologically, economic evaluations primarily answer questions about the relative value of an intervention compared to others. This is a related, but different question than the one addressed in this review. While economic evaluations may contain useful economic information, we anticipated difficulties in ascertaining from the papers’ full-text the extent to which the comparator or the intervention(s) in economic evaluations were (or would become) part of current practice, which would have been of key interest given our aim to describe the current economic burden of disease. Pragmatically, the fast growing number of economic evaluations conducted in LMICs during the past decade would have overburdened the review process, as well as interpreting the results. Third, we excluded studies reporting the economic burden of CVD risk factors other than hypertension.

### Implications

Our results highlight that annual costs of CVD care significantly exceed health expenditure per capita in most LMICs, raising the issues of financial protection for CVD patients and, more broadly, of health financing sustainability. Health financing reform as part of progress towards universal health coverage will have to mean more than mobilising additional funding and managing transition from donor support; it will have to strengthen the role of pre-paid contributions, effective pooling and strategic purchasing if health systems are to cope with the rising burden of CVD and NCDs at large. Caution is warranted in relation to the magnitude of our findings, however. Pooling together economic estimates from such heterogeneous, poor quality studies can only offer an indication of the true magnitude of the burden. Country-specific results of higher methodological quality are needed to inform national planning and decision-making.

Our findings confirm those of previous studies suggesting that the current evidence on the economic burden of CVD in LMICs does not appear aligned with policy priorities in terms of volume, focus and methodological quality [37]. More economic research of better methodological quality needs to be conducted in areas with current gaps, specifically: i) in community and primary care settings; ii) incorporating an appropriate control group; iii) collecting data from multiple sites with a view to representativeness; and iv) incorporating the full economic consequences of CVD prevention and care, not merely direct medical costs. More broadly, the rationale of conducting cost studies, as are the majority of studies included in this review, will require an increasingly clear and strong justification. While these are useful to estimate the costs of CVD interventions and to quantify household-level economic burden, they have limited value for policy compared to national-level cost-of-illness studies. In countries where reliable cost estimates of CVD interventions are missing, good quality cost studies will likely continue to be necessary with a view to informing credible cost-of-illness and health sector planning exercises. In countries where cost estimates are already available, methodological quality will need to improve hand in hand with a shift in research designs towards answering questions of interest to national policy makers.

The main reasons for the scarcity and low quality economic data in LMICs on the impact of NCDs more broadly and of CVD specifically are well known. There is still insufficient capacity and ownership for health systems and health economics research in LMICs [38, 39]. Despite long-standing international interest in research capacity strengthening [40, 41] with some promising results [42, 43], good practice recommendations in this area are relatively recent [44–46]. Second, before their explicit inclusion in the Sustainable Development Goals (Target 3.4 “*reduce by one third premature mortality from NCDs through prevention and treatment, and promote mental health and wellbeing*”), NCDs received insufficient attention from national governments relative to the yet unfinished agenda of infectious diseases. From an international development perspective, NCDs have received a disproportionately (albeit increasing) small amount of donor funding relative to their burden compared to other global health areas, despite countries’ requests for funding NCD programmes [47]. Although donor support can and should improve in the future, national governments will still need to identify solutions to fund NCD interventions and reduce the current burden. In this context, better quality economic data on the burden of CVD are necessary for building strong investment cases to national health decision makers towards investing in CVD prevention and treatment. While acknowledging that economic considerations are just one type of consideration in policy decisions [39], they still need to be explicit and robust if they are to be considered at all.

## Conclusions

We assessed and synthesized the evidence of CVD and hypertension economic impact across a wide range of economic outcomes and study designs, thereby offering the first comprehensive and systematic picture of research in the field. The economic impact of CVD and hypertension appears to be substantial relative to current health expenditure levels. Further research into the economic impact of CVD and hypertension is needed in many LMICs to inform appropriate NCD policies, with due focus on improving the quality of research and aligning research outputs with demonstrated disease burden. More broadly, national-level studies with appropriate sample sizes and adequate incorporation of indirect costs need to replace small-scale, institutional, retrospective cost studies.

## Additional files

Additional file 1: Search strategies for the included databases. (DOCX 54 kb)

Additional file 2: Quality assessment tool. (DOCX 14 kb)

Additional file 3: Selected descriptive characteristics of included studies. (DOCX 208 kb)

Additional file 4: Data extracted from included studies. (XLSX 272 kb)

Additional file 5: Cost burden relative to total health expenditure per capita. (TIFF 3515 kb)

## Abbreviations

CVD: Cardiovascular disease
DALY: Disability-adjusted life-year
GDP: Gross domestic product
HICs: High-income countries
LMICs: Low- and middle-income countries
NCD: Non-communicable disease

## References

[1] Vos T, et al., Global, regional, and national incidence, prevalence, and years lived with disability for 328 diseases and injuries for 195 countries, 1990–2016: A systematic analysis for the global burden of disease study 2016. The Lancet. 2017;390(10100):1211–1259. 10.1016/S0140-6736(17)32154-2 PMC5605509 28919117

[2] Hay SI, et al., Global, regional, and national disability-adjusted life-years (DALYs) for 333 diseases and injuries and healthy life expectancy (HALE) for 195 countries and territories, 1990–2016: A systematic analysis for the global burden of disease study 2016. 2017. The Lancet. 2017;390(10100):1260–1344. 10.1016/S0140-6736(17)32130-X PMC5605707 28919118

[3] Naghavi M, et al., Global, regional, and national age-sex specific mortality for 264 causes of death, 1980–2016: A systematic analysis for the global burden of disease study 2016. 2017. The Lancet. 2017;390(10100):1151–1210. 10.1016/S0140-6736(17)32152-9 PMC5605883 28919116

[4] Alwan A, MacLean DR (2009). A review of non-communicable disease in low- and middle-income countries. International Health.

[5] GBD 2013 Mortality and Causes of Death Collaborators (2014). Global, regional, and national age-sex specific all-cause and cause-specific mortality for 240 causes of death, 1990–2013: A systematic analysis for the global burden of disease study 2013. The Lancet.

[6] Bovet P, Paccaud F (2012). Cardiovascular disease and the changing face of global public health: a focus on low and middle income countries. Public Health Rev.

[7] Baingana FK, Bos ER, Jamison DT (2006). Changing patterns of disease and mortality in Sub-Saharan Africa: an overview, in Disease and Mortality in Sub-Saharan Africa.

[8] World Health Organization and World Economic Forum (2011). From Burden to "Best Buys": Reducing the Economic Impact of Non-Communicable Diseases in Low- and Middle-Income Countries.

[9] World Health Organization (2013). Global action plan for the prevention and control of noncommunicable diseases 2013–2010.

[10] World Health Organization (2014). Global Status Report on noncommunicable diseases 2014 "Attaining the nine global noncommunicable diseases targets: a shared responsibility".

[11] Roth GA (2015). Estimates of global and regional premature cardiovascular mortality in 2025. Circulation.

[12] Yusuf S (2015). The world heart Federation's vision for worldwide cardiovascular disease prevention. Lancet.

[13] Khatib R, et al. Availability and affordability of cardiovascular disease medicines and their effect on use in high-income, middle-income, and low-income countries: an analysis of the PURE study data. The Lancet. 2016;387(10013):61–69. 10.1016/S0140-6736(15)00469-9 26498706

[14] Bloom DE (2011). The global economic burden of non-communicable diseases.

[15] World Health Organization (2011). Global status report on noncommunicable diseases 2010.

[16] Huffman MD (2011). A cross-sectional study of the microeconomic impact of cardiovascular disease hospitalization in four low- and middle-income countries. PLoS One.

[17] Suhrcke M (2006). Chronic disease: an economic perspective.

[18] Nikolic IA, Stanciole AE, Zaydman M (2011). Chronic Emergency: Why NCDs Matter.

[19] Ebrahim S (2013). Tackling non-communicable diseases in low- and middle-income countries: is the evidence from high-income countries all we need?. PLoS Med.

[20] Moher D (2009). Preferred reporting items for systematic reviews and meta-analyses: the PRISMA statement. BMJ.

[21] Mendis S, Puska P, Norrving B, World Health Organization (2011). Global Atlas on Cardiovascular Disease Prevention and Control.

[22] Husereau D, et al. Consolidated health economic evaluation reporting standards (CHEERS) statement. BMJ. 2013;346. https://www.bmj.com/content/346/bmj.f1049. 10.1136/bmj.f1049 23529982

[23] Shemilt I, Thomas J, Morciano M (2010). A web-based tool for adjusting costs to a specific target currency and price year. Evidence Policy.

[24] R Core Team. R: A language and environment for statistical computing. R Foundation for Statistical Computing, Vienna, Austria. 2017. http://www.R-project.org/.

[25] Gaziano TA (2009). The global cost of nonoptimal blood pressure. J Hypertens.

[26] Azambuja MIR (2008). Economic burden of severe cardiovascular diseases in Brazil: an estimate based on secondary data [Impacto econômico dos casos de doença cardiovascular grave no Brasil: uma estimativa baseada em dados secundários]. Arq Bras Cardiol.

[27] Bloom, D.E., et al. The Economic Impact of Non-Communicable Disease in China and India: Estimates, Projections, and Comparisons. 2013 2013//.

[28] Maharaj JC, Reddy M. Young stroke mortality in Fiji islands: an economic analysis of national human capital resource loss*.* ISRN neurology, 2012. 10.5402/2012/802785 PMC3388426 22778993

[29] Lakic D, Tasic L, Kos M (2014). Economic burden of cardiovascular diseases in Serbia [Kardiovaskularne bolesti u Srbiji - Ekonomski teret]. Vojnosanit Pregl.

[30] Cook C (2014). The annual global economic burden of heart failure. Int J Cardiol.

[31] Kontsevaya A, Kalinina A, Oganov R (2013). Economic burden of cardiovascular diseases in the Russian federation. Value Health Reg Issues.

[32] Calvo-Vargas CG (1998). Changes in the costs of antihypertensive medications in a developing country: a study in Mexico comparing 1990 and 1996. Am J Hypertens.

[33] Zhao JJ (2013). Status and costs of primary prevention for ischemic stroke in China. J Clin Neurosci.

[34] Alam K, Mahal A (2014). Economic impacts of health shocks on households in low and middle income countries: a review of the literature. Glob Health.

[35] Brouwer ED (2015). Provider costs for prevention and treatment of cardiovascular and related conditions in low- and middle-income countries: a systematic review. BMC Public Health.

[36] Kankeu HT (2013). The financial burden from non-communicable diseases in low- and middle-income countries: a literature review. Health Res Policy Syst.

[37] Chisholm D (2010). Economic impact of disease and injury: counting what matters. BMJ.

[38] Adam T (2011). Trends in health policy and systems research over the past decade: still too little capacity in low-income countries. PLoS One.

[39] Mills, A., Reflections on the development of health economics in low- and middle-income countries. Proc R Soc Lond B Biol Sci. 2014;281(1789):20140451. https://www.ncbi.nlm.nih.gov/pmc/articles/PMC4100502/pdf/rspb20140451.pdf. 10.1098/rspb.2014.0451 PMC4100502 25009059

[40] Nuyens Y (2005). No development without research: a challenge for capacity strengthening.

[41] Lansang MA, Dennis R (2004). Building capacity in health research in the developing world. Bull World Health Organ.

[42] Minja H (2011). Impact of Health Research capacity strengthening in low- and middle-income countries: the case of WHO/TDR Programmes. PLoS Negl Trop Dis.

[43] Käser M (2016). Research capacity strengthening in low and middle income countries – an evaluation of the WHO/TDR career development fellowship Programme. PLoS Negl Trop Dis.

[44] Gadsby EW (2011). Research capacity strengthening: donor approaches to improving and assessing its impact in low- and middle-income countries. Int J Health Plann Manag.

[45] ESSENCE on Health Research, Seven principles for strengthening research capacity in low- and middle-income countries: simple ideas in a complex world. 2014.

[46] Bates I (2014). A practical and systematic approach to organisational capacity strengthening for research in the health sector in Africa. Health Res Policy Syst.

[47] Nugent R (2015). Bilateral and multilateral financing for NCDs. Policy brief for the Working Group on how to realize governments' commitment to provide financing for NCD.

