# Supplementary material for: The economic burden of cardiovascular disease and hypertension in low- and middle-income countries: a systematic review
Source: BMC Public Health. 2018 Aug 6;18:975. doi: 10.1186/s12889-018-5806-x (PMC6090747; doi:10.1186/s12889-018-5806-x)
Supplement: Supplementary file 1 — Search strategies for the included databases. (DOCX 54 kb) [file 12889_2018_5806_MOESM1_ESM.docx]

# Additional file 1 Search strategies for the included databases

Database: Africa-Wide Information (EBSCO)

| **#** | **Search terms** |
| --- | --- |
| S1 | TI (economic N2 (model* or forecast* or simulat* or predict* or estimat*)) OR AB (economic N2 (model* or forecast* or simulat* or predict* or estimat*)) |
| S2 | TI (earning* or income or poverty or expense* or out-of-pocket or OOP or payment* or expenditure* or labo#r or workforce or productiv* or absenteeism or employment or employab*) OR AB (earning* or income or poverty or expense* or out-of-pocket or OOP or payment* or expenditure* or labo#r or workforce or productiv* or absenteeism or employment or employab*) |
| S3 | TI ((cost* OR burden OR impact OR consequence*) N3 (disease* OR disorder* OR NCD* OR illness OR economic)) OR AB ((cost* OR burden OR impact OR consequence*) N3 (disease* OR disorder* OR NCD* OR illness OR economic)) |
| S4 | TI (gross domestic product OR national income OR economic growth) OR AB (gross domestic product OR national income OR economic growth) |
| S5 | TI cost-of-illness OR AB cost-of-illness |
| S6 | S1 OR S2 OR S3 OR S4 OR S5 |
| S7 | TI ((Heart or vascular or blood or cardio-vascular or cardiovascular or cerebro-vascular or cerebrovascular or coronary) N2 (disease* or disorder* or affect* or syndrome)) OR AB ((Heart or vascular or blood or cardio-vascular or cardiovascular or cerebro-vascular or cerebrovascular or coronary) N2 (disease* or disorder* or affect* or syndrome)) |
| S8 | TI (((atrial or ventricular) N2 (fibrillat* or flutter)) or isch#em* or aneurysm* or stroke or angina or myocard* infarct* or embolism or thrombo* or hypertensi* or anti-hypertensi* or antihypertensi* or blood pressure) OR AB (((atrial or ventricular) N2 (fibrillat* or flutter)) or isch#em* or aneurysm* or stroke or angina or myocard* infarct* or embolism or thrombo* or hypertensi* or anti-hypertensi* or antihypertensi* or blood pressure) |
| S9 | TI (asthm* or COPD or (chronic obstructive N1 (pulmonary or lung))) OR AB (asthm* or COPD or (chronic obstructive N1 (pulmonary or lung))) |
| S10 | TI (Neoplasm* or cancer* or lymphom* or myelom* or leuk#em* or malignan* or melanom* or tumo#r* or neoplas*) OR AB (Neoplasm* or cancer* or lymphom* or myelom* or leuk#em* or malignan* or melanom* or tumo#r* or neoplas*) |
| S11 | TI (Diabet* or (insulin* N2 (defic* or resistan*)) or (glucose N2 (toleran* or intoleran*)) or insulindepend* or insulin-depend* or noninsulin-depend*) OR AB (Diabet* or (insulin* N2 (defic* or resistan*)) or (glucose N2 (toleran* or intoleran*)) or insulindepend* or insulin-depend* or noninsulin-depend*) |
| S12 | TI (musculoskeletal or (((arthrit* or osteoarthrit* or gout or rheumat*) N2 arthrit*) or back pain or neck pain)) OR AB (musculoskeletal or (((arthrit* or osteoarthrit* or gout or rheumat*) N2 arthrit*) or back pain or neck pain)) |
| S13 | TI (depression or ((depressive or affective or bipolar or unipolar) N2 disorder*)) OR AB (depression or ((depressive or affective or bipolar or unipolar) N2 disorder*)) |
| S14 | TI (schizophren* or (personality N3 disorder) or epileps* or epilept* or seizure* or Alzheimer* or dementia* anorex* or bulim* or (eat* N2 disorder*) or agoraphobia or phobi* or obsessive-compulsive or obsessive or compulsive or anxiety or Parkinson* or ((multiple or lateral) N2 sclerosis)) OR AB (schizophren* or (personality N3 disorder) or epileps* or epilept* or seizure* or Alzheimer* or dementia* anorex* or bulim* or (eat* N2 disorder*) or agoraphobia or phobi* or obsessive-compulsive or obsessive or compulsive or anxiety or Parkinson* or ((multiple or lateral) N2 sclerosis)) |
| S15 | TI (insomni* or (sleep N2 (depriv* or disorder*)) or migrain* or headache* or head ache) OR AB (insomni* or (sleep N2 (depriv* or disorder*)) or migrain* or headache* or head ache) |
| S16 | TI ((alcohol* or drug or amphetamine* or cocaine or marijuana or opioid* or tobacco or substance) N3 (syndrome* or addict* or withdraw* or disorder* or abus* or abstinen*)) OR AB ((alcohol* or drug or amphetamine* or cocaine or marijuana or opioid* or tobacco or substance) N3 (syndrome* or addict* or withdraw* or disorder* or abus* or abstinen*)) |
| S17 | S7 OR S8 OR S9 OR S10 OR S11 OR S12 OR S13 OR S14 OR S15 OR S16 |
| **S18** | **S6 AND S17** |

Database: EconLit (Ovid)

| **#** | **Search terms** |
| --- | --- |
| 1 | ((Heart or vascular or blood or cardiovascular or cerebrovascular or coronary) adj2 (disease* or disorder* or affect* or syndrome)).ti,ab. |
| 2 | (((atrial or ventricular) adj2 (fibrillat* or flutter)) or isch?em* or aneurysm* or stroke or angina or (myocard* adj2 infarct*) or embolism or thrombo* or hypertensi* or anti-hypertensi* or (blood adj pressure)).ti,ab. |
| 3 | 1 or 2 |
| 4 | (Asthm* or COPD or (chronic obstructive adj (pulmonary or lung))).ti,ab. |
| 5 | (((arthrit* or osteoarthrit* or gout or rheumat*) adj2 arthrit*) or low back pain or neck pain).ti,ab. |
| 6 | (Neoplasm* or cancer* or lymphom* or myelom* or leuk?em* or malignan* or melanom* or tumo?r* or neoplas*).ti,ab. |
| 7 | (Diabet* or (insulin* adj2 (defic* or resistan*)) or (glucose adj2 (toleran* or intoleran*)) or non?insulin?depend* or insulin?depend* or noninsulin-depend*).ti,ab. |
| 8 | (depression or ((depressive or affective or bipolar or unipolar) adj2 disorder*)).ti,ab. |
| 9 | (schizophren* or (personality adj3 disorder) or epilep* or seizure* or Alzheimer* or dementia* anorex* or bulim* or (eat* adj2 disorder*) or agoraphobia or phobi* or obsessive?compulsive or obsessive or compulsive or anxiety or Parkinson* or ((multiple or lateral) adj2 sclerosis)).ti,ab. |
| 10 | (insomni* or (sleep adj2 (depriv* or disorder*)) or migrain* or headache* or head ache)).ti,ab. |
| 11 | ((alcohol* or drug or amphetamine* or cocaine or marijuana or opioid* or tobacco or substance) adj3 (syndrome* or addict* or withdraw* or disorder* or abus* or abstinen*)).ti,ab. |
| 12 | 1 or 2 or 3 or 4 or 5 or 6 or 7 or 8 or 9 or 10 or 11 |
| 13 | (Afghanistan or Gambia or Myanmar or Bangladesh or Guinea or Nepal or Benin or Bissau or Niger or Burkina or Haiti or Rwanda or Burundi or Kenya or Leone or Cambodia or Korea or Somalia or "Central African Republic" or Kyrgyz or Sudan or Chad or Liberia or Tajikistan or Comoros or Madagascar or Tanzania or Congo or Malawi or Togo or Eritrea or Mali or Uganda or Ethiopia or Mozambique or Zimbabwe).ti,ab. |
| 14 | (Armenia or India or Samoa or Bhutan or Kiribati or Tome or Bolivia or Kosovo or Senegal or Cameroon or Lao* or Solomon or "Cape Verde" or Lesotho or "Sri Lanka" or Congo or Mauritania or Sudan or "Côte d'Ivoire" or Ivory Coast or Micronesia or Swaziland or Djibouti or Moldova or Syria* or Egypt or Mongolia or Timor or "El Salvador" or Morocco or Ukraine or Georgia or Nicaragua or Uzbekistan or Ghana or Nigeria or Vanuatu or Guatemala or Pakistan or Vietnam or Guyana or Papua or "West Bank" or Gaza or Honduras or Paraguay or Yemen or Indonesia or Philippines or Zambia).ti,ab. |
| 15 | (Angola or Fiji or Palau or Albania or Gabon or Panama or Algeria or Grenada or Peru or Samoa or Hungary or Romania or Argentina or Iran or Serbia or Azerbaijan or Iraq or Seychelles or Belarus or Jamaica or "South Africa" or Belize or Jordan or "St. Lucia" or Saint Lucia or Bosnia or Kazakhstan or "St. Vincent" or Saint Vincent or Botswana or Lebanon or Suriname or Brazil or Libya or Thailand or Bulgaria or Macedonia or Tonga or China or Malaysia or Tunisia or Colombia or Maldives or Turkey or "Costa Rica" or "Marshall Islands" or Turkmenistan or Cuba or Mauritius or Tuvalu or Dominica* or Mexico or Venezuela or Montenegro or Ecuador or Namibia).ti,ab. |
| 16 | ((((low or medium or middle or mid) adj income) or developing) adj countr*).ab,ti. |
| 17 | (Africa or ((Latin or South or Central) adj1 America) or Asia or Pacific or (East* adj1 Europe)).ti,ab. |
| 18 | 13 or 14 or 15 or 16 or 17 |
| 19 | 12 and 18 |
| **20** | **limit 19 to yr="1990 -Current"** |

Database: EMBASE Classic + EMBASE (Ovid)

| **#** | **Search terms** |
| --- | --- |
| 1 | exp Cardiovascular Diseases/ |
| 2 | ((Heart or vascular or blood or cardiovascular or cerebrovascular or coronary) adj2 (disease* or disorder* or affect* or syndrome)).ab,ti. |
| 3 | (((atrial or ventricular) adj2 (fibrillat* or flutter)) or isch?em* or aneurysm* or stroke or angina or (myocard* adj infarct*) or embolism or thrombo* or hypertensi* or anti?hypertensi* or (blood adj pressure)).ab,ti. |
| 4 | 1 or 2 or 3 |
| 5 | (Asthm* or COPD or (chronic obstructive adj (pulmonary or lung))).ab,ti. |
| 6 | exp Pulmonary Disease, Chronic Obstructive/ |
| 7 | exp Asthma/ |
| 8 | 5 or 6 or 7 |
| 9 | exp *back pain/ or exp *neck pain/ |
| 10 | exp Musculoskeletal Diseases/ |
| 11 | (((arthrit* or osteoarthrit* or gout or rheumat*) adj2 arthrit*) or back pain or neck pain).ab,ti. |
| 12 | 9 or 10 or 11 |
| 13 | exp Neoplasms/ |
| 14 | (Neoplasm* or cancer* or lymphom* or myelom* or leuk?em* or malignan* or melanom* or tumo?r* or neoplas*).ab,ti. |
| 15 | 13 or 14 |
| 16 | exp Diabetes Mellitus, Type 1/ or exp Diabetes Mellitus, Type 2/ or exp Diabetes Mellitus/ |
| 17 | (Diabet* or (insulin* adj2 (defic* or resistan*)) or (glucose adj2 (toleran* or intoleran*)) or insulindepend* or insulin-depend* or noninsulin-depend*).ti,ab. |
| 18 | 16 or 17 |
| 19 | exp *Mental Disorders/ |
| 20 | exp *Mental Health/ |
| 21 | exp *Neurology/ |
| 22 | exp neurodegenerative diseases/ or exp neuromuscular diseases/ or exp sleep disorders/ |
| 23 | (depression or ((depressive or affective or bipolar or unipolar) adj2 disorder*)).ab,ti. |
| 24 | (schizophren* or (personality adj3 disorder) or epileps* or epilept* or seizure* or Alzheimer* or dementia* anorex* or bulim* or (eat* adj2 disorder*) or agoraphobia or phobi* or obsessive?compulsive or obsessive or compulsive or anxiety or Parkinson* or ((multiple or lateral) adj2 sclerosis)).ab,ti. |
| 25 | (insomni* or (sleep adj2 (depriv* or disorder*)) or migrain* or headache* or head ache*).ab,ti. |
| 26 | ((alcohol* or drug or amphetamine* or cocaine or marijuana or opioid* or tobacco or substance) adj3 (syndrome* or addict* or withdraw* or disorder* or abus* or abstinen*)).ab,ti. |
| 27 | 19 or 20 or 21 or 22 or 23 or 24 or 25 or 26 |
| 28 | 2 or 3 or 5 or 11 or 14 or 17 or 23 or 24 or 25 or 26 |
| 29 | 4 or 8 or 12 or 15 or 18 or 27 |
| 30 | (Afghanistan or Gambia or Myanmar or Bangladesh or Guinea or Nepal or Benin or Bissau or Niger or Burkina or Haiti or Rwanda or Burundi or Kenya or Leone or Cambodia or Korea or Somalia or "Central African Republic" or Kyrgyz* or Sudan or Chad or Liberia or Tajikistan or Comoros or Madagascar or Tanzania or Congo or Malawi or Togo or Eritrea or Mali or Uganda or Ethiopia or Mozambique or Zimbabwe).ab,ti. |
| 31 | (Armenia or India or Samoa or Bhutan or Kiribati or Tome or Bolivia or Kosovo or Senegal or Cameroon or Lao* or Solomon or "Cape Verde" or Lesotho or "Sri Lanka" or Congo or Mauritania or Sudan or "Côte d'Ivoire" or "Ivory Coast" or Micronesia or Swaziland or Djibouti or Moldova or Syria* or Egypt or Mongolia or Timor or "El Salvador" or Morocco or Ukraine or Georgia or Nicaragua or Uzbekistan or Ghana or Nigeria or Vanuatu or Guatemala or Pakistan or Vietnam or Guyana or Papua or "West Bank" or Gaza or Honduras or Paraguay or Yemen or Indonesia or Philippines or Zambia).ab,ti. |
| 32 | (Angola or Fiji or Palau or Albania or Gabon or Panama or Algeria or Grenada or Peru or Samoa or Hungary or Romania or Argentina or Iran or Serbia or Azerbaijan or Iraq or Seychelles or Belarus or Jamaica or "South Africa" or Belize or Jordan or "St. Lucia" or "Saint Lucia" or Bosnia or Kazakhstan or "St. Vincent" or "Saint Vincent" or Botswana or Lebanon or Suriname or Brazil or Libya or Thailand or Bulgaria or Macedonia or Tonga or China or Malaysia or Tunisia or Colombia or Maldives or Turkey or "Costa Rica" or "Marshall Islands" or Turkmenistan or Cuba or Mauritius or Tuvalu or Dominica* or Mexico or Venezuela or Montenegro or Ecuador or Namibia).ab,ti. |
| 33 | exp africa/ or exp caribbean region/ or exp central america/ or exp latin america/ or exp mexico/ or exp south america/ or exp asia/ or exp europe, eastern/ or exp transcaucasia/ or exp pacific islands/ |
| 34 | exp Developing Countries/ |
| 35 | ((((low or medium or middle or mid) adj income) or developing) adj countr*).ab,ti. |
| 36 | 30 or 31 or 32 or 33 or 34 or 35 |
| 37 | exp Economics/ |
| 38 | exp "cost of illness"/ |
| 39 | exp Models, Economic/ |
| 40 | exp Efficiency/ |
| 41 | exp Income/ |
| 42 | exp Poverty/ |
| 43 | exp Employment/ |
| 44 | (economic adj2 (model* or forecast* or simulat* or predict* or estimat*)).ab,ti. |
| 45 | (earning* or income or poverty or expense* or out-of-pocket or OOP or payment* or expenditure* or labo?r or workforce or productiv* or absenteeism or employment or employab*).ti,ab. |
| 46 | ((cost* or burden or impact or consequence*) adj3 (disease* or disorder* or NCD* or illness or economic)).ab,ti. |
| 47 | (gross domestic product or national income or economic growth).ab,ti. |
| 48 | 37 or 38 or 39 or 40 or 41 or 42 or 43 or 44 or 45 or 46 or 47 |
| 49 | 29 and 36 and 48 |
| 50 | limit 49 to yr="1990 -Current" |
| 51 | (animal not (human and animal)).sh. |
| 52 | (rat or rats or rodent* or mouse or mice or murine or dog or dogs or canine* or cat or cats or feline* or rabbit or rabbits or pig or pigs or porcine or swine or sheep or ovine* or guinea pig*).ti. |
| 53 | 51 or 52 |
| 54 | 50 not 53 |
| 55 | (comment or editorial or letter).pt. |
| **56** | **54 not 55** |

Database: MEDLINE (Ovid)

| **#** | **Search terms** |
| --- | --- |
| 1 | exp Cardiovascular Diseases/ |
| 2 | ((Heart or vascular or blood or cardiovascular or cerebrovascular or coronary) adj2 (disease* or disorder* or affect* or syndrome)).ab,ti. |
| 3 | (((atrial or ventricular) adj2 (fibrillat* or flutter)) or isch?em* or aneurysm* or stroke or angina or (myocard* adj2 infarct*) or embolism or thrombo* or hypertensi* or anti-hypertensi* or (blood adj pressure)).ab,ti. |
| 4 | 1 or 2 or 3 |
| 5 | (Asthm* or COPD or ((chronic obstructive) adj (pulmonary or lung)).ab,ti. |
| 6 | exp Pulmonary Disease, Chronic Obstructive/ |
| 7 | exp Asthma/ |
| 8 | 5 or 6 or 7 |
| 9 | exp *back pain/ or exp *neck pain/ |
| 10 | exp Musculoskeletal Diseases/ |
| 11 | (((arthrit* or osteoarthrit* or gout or rheumat*) adj2 arthrit*) or back pain or neck pain).ab,ti. |
| 12 | 9 or 10 or 11 |
| 13 | exp Neoplasms/ |
| 14 | (Neoplasm* or cancer* or lymphom* or myelom* or leuk?em* or malignan* or melanom* or tumo?r* or neoplas*).ab,ti. |
| 15 | 13 or 14 |
| 16 | exp Diabetes Mellitus, Type 1/ or exp Diabetes Mellitus, Type 2/ or exp Diabetes Mellitus/ |
| 17 | (Diabet* or (insulin* adj2 (defic* or resistan*)) or (glucose adj2 (toleran* or intoleran*)) or insulindepend* or insulin-depend* or noninsulin-depend*)ti,ab. |
| 18 | 16 or 17 |
| 19 | exp Mental Disorders/ |
| 20 | exp Mental Health/ |
| 21 | exp Neurology/ |
| 22 | exp neurodegenerative diseases/ or exp neuromuscular diseases/ or exp sleep disorders/ |
| 23 | (depression or ((depressive or affective or bipolar or unipolar) adj2 disorder*)).ab,ti. |
| 24 | (schizophren* or (personality adj3 disorder) or epilep* or seizure* or Alzheimer* or dementia* anorex* or bulim* or (eat* adj2 disorder*) or agoraphobia or phobi* or obsessive?compulsive or obsessive or compulsive or anxiety or Parkinson* or ((multiple or lateral) adj2 sclerosis)).ab,ti. |
| 25 | (insomni* or (sleep adj2 (depriv* or disorder*)) or migrain* or headache* or head ache).ab,ti. |
| 26 | ((alcohol* or drug or amphetamine* or cocaine or marijuana or opioid* or tobacco or substance) adj3 (syndrome* or addict* or withdraw* or disorder* or abus* or abstinen*)).ab,ti. |
| 27 | 19 or 20 or 21 or 22 or 23 or 24 or 25 or 26 |
| 28 | 2 or 3 or 5 or 11 or 14 or 17 or 23 or 24 or 25 or 26 |
| 29 | 4 or 8 or 12 or 15 or 18 or 27 |
| 30 | (Afghanistan or Gambia or Myanmar or Bangladesh or Guinea or Nepal or Benin or Bissau or Niger or Burkina or Haiti or Rwanda or Burundi or Kenya or Leone or Cambodia or Korea or Somalia or "Central African Republic" or Kyrgyz* or Sudan or Chad or Liberia or Tajikistan or Comoros or Madagascar or Tanzania or Congo or Malawi or Togo or Eritrea or Mali or Uganda or Ethiopia or Mozambique or Zimbabwe).ab,ti. |
| 31 | (Armenia or India or Samoa or Bhutan or Kiribati or Tome or Bolivia or Kosovo or Senegal or Cameroon or Lao* or Solomon or "Cape Verde" or Lesotho or "Sri Lanka" or Congo or Mauritania or Sudan or "Côte d'Ivoire" or "Ivory Coast" or Micronesia or Swaziland or Djibouti or Moldova or Syria* or Egypt or Mongolia or Timor or "El Salvador" or Morocco or Ukraine or Georgia or Nicaragua or Uzbekistan or Ghana or Nigeria or Vanuatu or Guatemala or Pakistan or Vietnam or Guyana or Papua or "West Bank" or Gaza or Honduras or Paraguay or Yemen or Indonesia or Philippines or Zambia).ab,ti. |
| 32 | (Angola or Fiji or Palau or Albania or Gabon or Panama or Algeria or Grenada or Peru or Samoa or Hungary or Romania or Argentina or Iran or Serbia or Azerbaijan or Iraq or Seychelles or Belarus or Jamaica or "South Africa" or Belize or Jordan or "St. Lucia" or "Saint Lucia" or Bosnia or Kazakhstan or "St. Vincent" or "Saint Vincent" or Botswana or Lebanon or Suriname or Brazil or Libya or Thailand or Bulgaria or Macedonia or Tonga or China or Malaysia or Tunisia or Colombia or Maldives or Turkey or "Costa Rica" or "Marshall Islands" or Turkmenistan or Cuba or Mauritius or Tuvalu or Dominica* or Mexico or Venezuela or Montenegro or Ecuador or Namibia).ab,ti. |
| 33 | exp africa/ or exp caribbean region/ or exp central america/ or exp latin america/ or exp mexico/ or exp south america/ or exp asia/ or exp europe, eastern/ or exp transcaucasia/ or exp pacific islands/ |
| 34 | exp Developing Countries/ |
| 35 | ((((low or medium or middle or mid) adj income) or developing) adj countr*).ab,ti. |
| 36 | 30 or 31 or 32 or 33 or 34 or 35 |
| 37 | exp Economics/ |
| 38 | exp "cost of illness"/ |
| 39 | exp Models, Economic/ |
| 40 | exp Efficiency/ |
| 41 | exp Income/ |
| 42 | exp Poverty/ |
| 43 | exp Employment/ |
| 44 | (economic adj2 (model* or forecast* or simulat* or predict* or estimat*)).ab,ti. |
| 45 | (earning* or income or poverty or expense* or out-of-pocket or OOP or payment* or expenditure* or labo?r or workforce or productiv* or absenteeism or employment or employab*).ti,ab. |
| 46 | ((cost* or burden or impact or consequence*) adj3 (disease* or disorder* or NCD* or illness or economic)).ab,ti. |
| 47 | (gross domestic product or national income or economic growth).ab,ti. |
| 48 | 37 or 38 or 39 or 40 or 41 or 42 or 43 or 44 or 45 or 46 or 47 |
| 49 | 29 and 36 and 48 |
| 50 | limit 49 to yr="1990 -Current" |
| 51 | (animals not (humans and animals)).sh. |
| 52 | (rat or rats or rodent* or mouse or mice or murine or dog or dogs or canine* or cat or cats or feline* or rabbit or rabbits or pig or pigs or porcine or swine or sheep or ovine* or guinea pig*).ti. |
| 53 | 51 or 52 |
| 54 | 50 not 53 |
| 55 | (comment or editorial or letter).pt. |
| **56** | **54 not 55** |

Database: COCHRANE (All Databases)

| **#** | **Search terms** |
| --- | --- |
| 1 | MeSH descriptor: [Cardiovascular Diseases] explode all trees |
| 2 | ((Heart or vascular or blood or cardiovascular or cerebrovascular or coronary) near/2 (disease* or disorder* or affect* or syndrome)) |
| 3 | (((atrial or ventricular) near/2 (fibrillat* or flutter)) or isch?em* or aneurysm* or stroke or angina or (myocard* near/1 infarct*) or embolism or thrombo* or hypertensi* or anti?hypertensi* or (blood near/1 pressure)) |
| 4 | #1 or #2 or #3 |
| 5 | MeSH descriptor: [Diabetes Mellitus] explode all trees |
| 6 | (Diabet* or (insulin* near/2 (defic* or resistan*)) or (glucose near/2 (toleran* or intoleran*)) or insulin-depend* or insulindepend* or noninsulin-depend*) |
| 7 | #5 or #6 |
| 8 | MeSH descriptor: [Pulmonary Disease, Chronic Obstructive] explode all trees |
| 9 | MeSH descriptor: [Asthma] 3 tree(s) exploded |
| 10 | (Asthm* or COPD or ((chronic obstructive) near/1 (pulmonary or lung))) |
| 11 | #8 or #9 or #10 |
| 12 | MeSH descriptor: [Musculoskeletal Diseases] explode all trees |
| 13 | MeSH descriptor: [Back Pain] explode all trees |
| 14 | MeSH descriptor: [Neck Pain] explode all trees |
| 15 | (((arthrit* or osteoarthrit* or gout or rheumat*) near/2 arthrit*) or back pain or neck pain) |
| 16 | #12 or #13 or #14 or #15 |
| 17 | MeSH descriptor: [Neoplasms] explode all trees |
| 18 | (Neoplasm* or cancer* or lymphom* or myelom* or leuk?em* or malignan* or melanom* or tumo?r* or neoplas*) |
| 19 | #17 or #18 |
| 20 | MeSH descriptor: [Mental Disorders] explode all trees |
| 21 | MeSH descriptor: [Mental Health] explode all trees |
| 22 | MeSH descriptor: [Neurodegenerative Diseases] explode all trees |
| 23 | MeSH descriptor: [Neuromuscular Diseases] explode all trees |
| 24 | MeSH descriptor: [Sleep Disorders] explode all trees |
| 25 | (depression or ((depressive or affective or bipolar or unipolar) near/2 disorder*)) |
| 26 | (schizophren* or (personality near/3 disorder) or epileps* or epilept* or seizure* or Alzheimer* or dementia* anorex* or bulim* or (eat* near/2 disorder*) or agoraphobia or phobi* or obsessive?compulsive or obsessive or compulsive or anxiety or Parkinson* or ((multiple or lateral) near/2 sclerosis)) |
| 27 | (insomni* or (sleep near/2 (depriv* or disorder*)) or migrain* or headache* or head ache) |
| 28 | ((alcohol* or drug or amphetamine* or cocaine or marijuana or opioid* or tobacco or substance) near/3 (syndrome* or addict* or withdraw* or disorder* or abus* or abstinen*)) |
| 29 | #20 or #21 or #22 or #23 or #24 or #25 or #26 or #27 or #28 |
| 30 | #4 or #7 or #11 or #16 or #19 or #29 |
| 31 | MeSH descriptor: [Africa] explode all trees |
| 32 | MeSH descriptor: [Caribbean Region] explode all trees |
| 33 | MeSH descriptor: [Central America] explode all trees |
| 34 | MeSH descriptor: [Latin America] explode all trees |
| 35 | MeSH descriptor: [South America] explode all trees |
| 36 | MeSH descriptor: [Europe, Eastern] explode all trees |
| 37 | MeSH descriptor: [Transcaucasia] explode all trees |
| 38 | MeSH descriptor: [Pacific Islands] explode all trees |
| 39 | MeSH descriptor: [Developing Countries] explode all trees |
| 40 | (Afghanistan or Gambia or Myanmar or Bangladesh or Guinea or Nepal or Benin or Bissau or Niger or Burkina or Haiti or Rwanda or Burundi or Kenya or Leone or Cambodia or Korea or Somalia or "Central African Republic" or Kyrgyz* or Sudan or Chad or Liberia or Tajikistan or Comoros or Madagascar or Tanzania or Congo or Malawi or Togo or Eritrea or Mali or Uganda or Ethiopia or Mozambique or Zimbabwe) |
| 41 | (Armenia or India or Samoa or Bhutan or Kiribati or Tome or Bolivia or Kosovo or Senegal or Cameroon or Lao* or Solomon or "Cape Verde" or Lesotho or "Sri Lanka" or Congo or Mauritania or Sudan or "Côte d'Ivoire" or "Ivory Coast" or Micronesia or Swaziland or Djibouti or Moldova or Syria* or Egypt or Mongolia or Timor or "El Salvador" or Morocco or Ukraine or Georgia or Nicaragua or Uzbekistan or Ghana or Nigeria or Vanuatu or Guatemala or Pakistan or Vietnam or Guyana or Papua or "West Bank" or Gaza or Honduras or Paraguay or Yemen or Indonesia or Philippines or Zambia) |
| 42 | (Angola or Fiji or Palau or Albania or Gabon or Panama or Algeria or Grenada or Peru or Samoa or Hungary or Romania or Argentina or Iran or Serbia or Azerbaijan or Iraq or Seychelles or Belarus or Jamaica or South Africa or Belize or Jordan or St. Lucia or Saint Lucia or Bosnia or Kazakhstan or St. Vincent or Saint Vincent or Botswana or Lebanon or Suriname or Brazil or Libya or Thailand or Bulgaria or Macedonia or Tonga or China or Malaysia or Tunisia or Colombia or Maldives or Turkey or Costa Rica or Marshall Islands or Turkmenistan or Cuba or Mauritius or Tuvalu or Dominica* or Mexico or Venezuela or Montenegro or Ecuador or Namibia) |
| 43 | ((((low or medium or middle or mid) near/1 income) or developing) near/1 countr*) |
| 44 | #31 or #32 or #33 or #34 or #35 or #36 or #37 or #38 or #39 or #40 or #41 or #42 or #43 |
| 45 | MeSH descriptor: [Economics] explode all trees |
| 46 | MeSH descriptor: [Cost of Illness] explode all trees |
| 47 | MeSH descriptor: [Models, Economic] explode all trees |
| 48 | MeSH descriptor: [Efficiency] explode all trees |
| 49 | MeSH descriptor: [Income] explode all trees |
| 50 | MeSH descriptor: [Poverty] explode all trees |
| 51 | MeSH descriptor: [Employment] explode all trees |
| 52 | (economic near/2 (model* or forecast* or simulat* or predict* or estimat*)) |
| 53 | (earning* or income or poverty or expense* or out-of-pocket or OOP or payment* or expenditure* or labo?r or workforce or productiv* or absenteeism or employment or employab*) |
| 54 | ((cost* or burden or impact or consequence*) near/3 (disease* or disorder* or NCD* or illness or economic)) |
| 55 | (gross domestic product or national income or economic growth) |
| 56 | #45 or #46 or #47 or #48 or #49 or #50 or #51 or #52 or #53 or #54 or #55 |
| **57** | **#30 and #44 and #56** |

Database: PsycInfo

| **#** | **Search terms** |
| --- | --- |
| 1 | exp Cardiovascular Disorders/ |
| 2 | ((Heart or vascular or blood or cardiovascular or cerebrovascular or coronary) adj2 (disease* or disorder* or affect* or syndrome)).ab,ti. |
| 3 | (((atrial or ventricular) adj2 (fibrillat* or flutter)) or isch?em* or aneurysm* or stroke or angina or (myocard* adj2 infarct*) or embolism or thrombo* or hypertensi* or anti?hypertensi* or (blood adj pressure)).ab,ti. |
| 4 | 1 or 2 or 3 |
| 5 | (Asthm* or COPD or (chronic obstructive adj (pulmonary or lung))).ab,ti. |
| 6 | exp Chronic Obstructive Pulmonary Disease/ |
| 7 | exp Asthma/ |
| 8 | 5 or 6 or 7 |
| 9 | exp *back pain/ or exp *neck pain/ |
| 10 | exp Musculoskeletal Disorders/ |
| 11 | (((arthrit* or osteoarthrit* or gout or rheumat*) adj2 arthrit*) or back pain or neck pain).ab,ti. |
| 12 | 9 or 10 or 11 |
| 13 | exp Neoplasms/ |
| 14 | (Neoplasm* or cancer* or lymphom* or myelom* or leuk?em* or malignan* or melanom* or tumo?r* or neoplas*).ab,ti. |
| 15 | 13 or 14 |
| 16 | exp Diabetes Mellitus, Type 1/ or exp Diabetes Mellitus, Type 2/ or exp Diabetes Mellitus/ |
| 17 | (Diabet* or (insulin* adj2 (defic* or resistan*)) or (glucose adj2 (toleran* or intoleran*)) or insulin-depend* or insulin*depend* or noninsulin-depend*).ti,ab. |
| 18 | 16 or 17 |
| 19 | exp Mental Disorders/ |
| 20 | exp Mental Health/ |
| 21 | exp Neurology/ |
| 22 | exp neurodegenerative diseases/ or exp neuromuscular diseases/ or exp sleep disorders/ |
| 23 | (depression or ((depressive or affective or bipolar or unipolar) adj2 disorder*)).ab,ti. |
| 24 | (schizophren* or (personality adj3 disorder) or epilep* or seizure* or Alzheimer* or dementia* or anorex* or bulim* or (eat* adj2 disorder*) or agoraphobia or phobi* or obsessive?compulsive or obsessive or compulsive or anxiety or Parkinson* or ((multiple or lateral) adj2 sclerosis)).ab,ti. |
| 25 | (insomni* or (sleep adj2 (depriv* or disorder*)) or migrain* or headache* or head ache).ab,ti. |
| 26 | ((alcohol* or drug or amphetamine* or cocaine or marijuana or opioid* or tobacco or substance) adj3 (syndrome* or addict* or withdraw* or disorder* or abus* or abstinen*)).ab,ti. |
| 27 | 19 or 20 or 21 or 22 or 23 or 24 or 25 or 26 |
| 28 | 2 or 3 or 5 or 11 or 14 or 17 or 23 or 24 or 25 or 26 |
| 29 | 4 or 8 or 12 or 15 or 18 or 27 |
| 30 | (Afghanistan or Gambia or Myanmar or Bangladesh or Guinea or Nepal or Benin or Bissau or Niger or Burkina or Haiti or Rwanda or Burundi or Kenya or Leone or Cambodia or Korea or Somalia or "Central African Republic" or Kyrgyz* or Sudan or Chad or Liberia or Tajikistan or Comoros or Madagascar or Tanzania or Congo or Malawi or Togo or Eritrea or Mali or Uganda or Ethiopia or Mozambique or Zimbabwe).ab,ti. |
| 31 | (Armenia or India or Samoa or Bhutan or Kiribati or Tome or Bolivia or Kosovo or Senegal or Cameroon or Lao* or Solomon or "Cape Verde" or Lesotho or "Sri Lanka" or Congo or Mauritania or Sudan or "Côte d'Ivoire" or "Ivory Coast" or Micronesia or Swaziland or Djibouti or Moldova or Syria* or Egypt or Mongolia or Timor or "El Salvador" or Morocco or Ukraine or Georgia or Nicaragua or Uzbekistan or Ghana or Nigeria or Vanuatu or Guatemala or Pakistan or Vietnam or Guyana or Papua or "West Bank" or Gaza or Honduras or Paraguay or Yemen or Indonesia or Philippines or Zambia).ab,ti. |
| 32 | (Angola or Fiji or Palau or Albania or Gabon or Panama or Algeria or Grenada or Peru or Samoa or Hungary or Romania or Argentina or Iran or Serbia or Azerbaijan or Iraq or Seychelles or Belarus or Jamaica or "South Africa" or Belize or Jordan or "St. Lucia" or "Saint Lucia" or Bosnia or Kazakhstan or "St. Vincent" or "Saint Vincent" or Botswana or Lebanon or Suriname or Brazil or Libya or Thailand or Bulgaria or Macedonia or Tonga or China or Malaysia or Tunisia or Colombia or Maldives or Turkey or "Costa Rica" or "Marshall Islands" or Turkmenistan or Cuba or Mauritius or Tuvalu or Dominica* or Mexico or Venezuela or Montenegro or Ecuador or Namibia).ab,ti. |
| 33 | exp Developing Countries/ |
| 34 | ((((low or medium or middle or mid) adj income) or developing) adj countr*).ab,ti. |
| 35 | 30 or 31 or 32 or 33 or 34 |
| 36 | exp Economics/ |
| 37 | exp Efficiency/ |
| 38 | exp Poverty/ |
| 39 | exp Employment/ |
| 40 | (economic adj2 (model* or forecast* or simulat* or predict* or estimat*)).ab,ti. |
| 41 | (earning* or income or poverty or expense* or out-of-pocket or OOP or payment* or expenditure* or labo?r or workforce or productiv* or absenteeism or employment or employab*).ti,ab. |
| 42 | ((cost* or burden or impact or consequence*) adj3 (disease* or disorder* or NCD* or illness or economic)).ab,ti. |
| 43 | (gross domestic product or national income or economic growth).ab,ti. |
| 44 | 36 or 37 or 38 or 39 or 40 or 41 or 42 or 43 |
| 45 | 29 and 35 and 44 |
| 46 | (animals not (humans and animals)).sh. |
| 47 | (rat or rats or rodent* or mouse or mice or murine or dog or dogs or canine* or cat or cats or feline* or rabbit or rabbits or pig or pigs or porcine or swine or sheep or ovine* or guinea pig*).ti. |
| 48 | 46 or 47 |
| **49** | **45 not 48** |

Database: Global Health (Ovid)

| **#** | **Search terms** |
| --- | --- |
| 1 | exp cardiovascular diseases/ |
| 2 | ((Heart or vascular or blood or cardiovascular or cerebrovascular or coronary) adj2 (disease* or disorder* or affect* or syndrome)).ab,ti. |
| 3 | (((atrial or ventricular) adj2 (fibrillat* or flutter)) or isch?em* or aneurysm* or stroke or angina or (myocard* adj2 infarct*) or embolism or thrombo* or hypertensi* or anti?hypertensi* or (blood adj pressure)).ab,ti. |
| 4 | 1 or 2 or 3 |
| 5 | (Asthm* or COPD or ((chronic obstructive) adj (pulmonary or lung))).ab,ti. |
| 6 | exp asthma/ |
| 7 | exp chronic obstructive pulmonary disease/ |
| 8 | 5 or 6 or 7 |
| 9 | (((arthrit* or osteoarthrit* or gout or rheumat*) adj2 arthrit*) or back pain or neck pain).ab,ti. |
| 10 | exp neoplasms/ |
| 11 | (Neoplasm* or cancer* or lymphom* or myelom* or leuk?em* or malignan* or melanom* or tumo$r* or neoplas*).ab,ti. |
| 12 | 10 or 11 |
| 13 | (Diabet* or (insulin* adj2 (defic* or resistan*)) or (glucose adj2 (toleran* or intoleran*)) or insulindepend* or insulin-depend* or noninsulin-depend*).ab,ti. |
| 14 | exp type 1 diabetes/ or exp diabetes mellitus/ or exp type 2 diabetes/ |
| 15 | 13 or 14 |
| 16 | exp mental disorders/ |
| 17 | exp mental health/ |
| 18 | exp neurology/ |
| 19 | exp neuromuscular diseases/ |
| 20 | (depression or ((depressive or affective or bipolar or unipolar) adj2 disorder*)).ab,ti. |
| 21 | (schizophren* or (personality adj3 disorder) or epileps* or epilept* or seizure* or Alzheimer* or dementia* anorex* or bulim* or (eat* adj2 disorder*) or agoraphobia or phobi* or obsessive?compulsive or obsessive or compulsive or anxiety or Parkinson* or ((multiple or lateral) adj2 sclerosis)).ab,ti. |
| 22 | (insomni* or (sleep adj2 (depriv* or disorder*)) or migrain* or headache* or head ache).ab,ti. |
| 23 | ((alcohol* or drug or amphetamine* or cocaine or marijuana or opioid* or tobacco or substance) adj3 (syndrome* or addict* or withdraw* or disorder* or abus* or abstinen*)).ab,ti. |
| 24 | 16 or 17 or 18 or 19 or 20 or 21 or 22 or 23 |
| 25 | 4 or 8 or 9 or 12 or 15 or 24 |
| 26 | (Afghanistan or Gambia or Myanmar or Bangladesh or Guinea or Nepal or Benin or Bissau or Niger or Burkina or Haiti or Rwanda or Burundi or Kenya or Leone or Cambodia or Korea or Somalia or "Central African Republic" or Kyrgyz* or Sudan or Chad or Liberia or Tajikistan or Comoros or Madagascar or Tanzania or Congo or Malawi or Togo or Eritrea or Mali or Uganda or Ethiopia or Mozambique or Zimbabwe).ti,ab. |
| 27 | (Armenia or India or Samoa or Bhutan or Kiribati or Tome or Bolivia or Kosovo or Senegal or Cameroon or Lao* or Solomon or "Cape Verde" or Lesotho or "Sri Lanka" or Congo or Mauritania or Sudan or "Côte d'Ivoire" or Ivory Coast or Micronesia or Swaziland or Djibouti or Moldova or Syria* or Egypt or Mongolia or Timor or "El Salvador" or Morocco or Ukraine or Georgia or Nicaragua or Uzbekistan or Ghana or Nigeria or Vanuatu or Guatemala or Pakistan or Vietnam or Guyana or Papua or "West Bank" or Gaza or Honduras or Paraguay or Yemen or Indonesia or Philippines or Zambia).ab,ti. |
| 28 | (Angola or Fiji or Palau or Albania or Gabon or Panama or Algeria or Grenada or Peru or Samoa or Hungary or Romania or Argentina or Iran or Serbia or Azerbaijan or Iraq or Seychelles or Belarus or Jamaica or "South Africa" or Belize or Jordan or "St. Lucia" or Saint Lucia or Bosnia or Kazakhstan or "St. Vincent" or Saint Vincent or Botswana or Lebanon or Suriname or Brazil or Libya or Thailand or Bulgaria or Macedonia or Tonga or China or Malaysia or Tunisia or Colombia or Maldives or Turkey or "Costa Rica" or "Marshall Islands" or Turkmenistan or Cuba or Mauritius or Tuvalu or Dominica* or Mexico or Venezuela or Montenegro or Ecuador or Namibia).ab,ti. |
| 29 | ((((low or medium or middle or mid) adj income) or developing) adj countr*).ab,ti. |
| 30 | (Africa or ((Latin or South or Central) adj1 America) or Asia or Pacific or (East* adj1 Europe)).ti,ab. |
| 31 | exp Developing Countries/ |
| 32 | 26 or 27 or 28 or 29 or 30 or 31 |
| 33 | exp economics/ |
| 34 | utilization/ or exp health care utilization/ |
| 35 | exp income/ |
| 36 | exp poverty/ |
| 37 | exp employment/ |
| 38 | exp efficiency/ |
| 39 | (economic adj2 (model* or forecast* or simulat* or predict* or estimat*)).ab,ti. |
| 40 | (earning* or income or poverty or expense* or out-of-pocket or OOP or payment* or expenditure* or labo?r or workforce or productiv* or absenteeism or employment or employab*).ti,ab. |
| 41 | ((cost* or burden or impact or consequence*) adj3 (disease* or disorder* or NCD* or illness or economic)).ab,ti. |
| 42 | (gross domestic product or national income or economic growth).ab,ti. |
| 43 | 33 or 34 or 35 or 36 or 37 or 38 or 39 or 40 or 41 or 42 |
| 44 | 25 and 32 and 43 |
| 45 | limit 44 to yr="1990 -Current" |
| 46 | (correspondence or editorial).pt. |
| 47 | 45 not 46 |
| 48 | (rat or rats or rodent* or mouse or mice or murine or dog or dogs or canine* or cat or cats or feline* or rabbit or rabbits or pig or pigs or porcine or swine or sheep or ovine* or guinea pig*).ti. |
| **49** | **47 not 48** |

Database: LILACS

| **#** | **Search term** |
| --- | --- |
| 1 | earning$ or income or poverty or expense$ or out-of-pocket or OOP or payment$ or expenditure$ or labor or labour or workforce or productiv$ or absenteeism or employment or employab$ or gross domestic product or national income or economic growth or cost of illness or cost-of-illness or burden of illness or burden-of-illness or economic burden or economic consequences or economic model [Words] |
| 2 | "CARDIOVASCULAR DISEASES/" or "ASTHMA" or "CHRONIC OBSTRUCTIVE PULMONARY DISEASE/" or "NEOPLASM" or "DIABETES" or "MUSCULOSKELETAL DISEASES/" or "MENTAL DISORDERS" or "MENTAL HEALTH" or "NEUROLOGY/" or "NEUROMUSCULAR DISEASES/" or "BACK PAIN/" or "NECK PAIN/" [Words] |
| 3 | heart or vascular or blood or cardiovascular or cerebrovascular or coronary or atrial or ventricular or fibrillat$ or myocard$ or hypertensi$ or stroke or angina or aneurysm or asthm$ or COPD or chronic obstructive or arthrit$ or gout$ or rheumat$ or diabet$ or depressi$ or bipolar or unipolar or schizophren$ or epilep$ or seizure or dementia or Parkinson$ or anorex$ or bulim$ or obsessive or compulsive or anxiety or multiple sclerosis or insomni$ or migrain$ or headache or head ache or addict$ or withdraw$ or abstinen$ or non-communicable or noncommunicable or NCD$ [Words] |
| 4 | 2 or 3 |
| **5** | **1 and 4** |

Database: MedCarib

| **#** | **Search term** |
| --- | --- |
| 1 | earning$ or income or poverty or expense$ or out-of-pocket or OOP or payment$ or expenditure$ or labor or labour or workforce or productiv$ or absenteeism or employment or employab$ or gross domestic product or national income or economic growth or cost of illness or cost-of-illness or burden of illness or burden-of-illness or economic burden or economic consequences or economic model [Words] |
| 2 | "CARDIOVASCULAR DISEASES/" or "ASTHMA" or "CHRONIC OBSTRUCTIVE PULMONARY DISEASE/" or "NEOPLASM" or "DIABETES" or "MUSCULOSKELETAL DISEASES/" or "MENTAL DISORDERS" or "MENTAL HEALTH" or "NEUROLOGY/" or "NEUROMUSCULAR DISEASES/" or "BACK PAIN/" or "NECK PAIN/" [Words] |
| 3 | heart or vascular or blood or cardiovascular or cerebrovascular or coronary or atrial or ventricular or fibrillat$ or myocard$ or hypertensi$ or stroke or angina or aneurysm or asthm$ or COPD or chronic obstructive or arthrit$ or gout$ or rheumat$ or diabet$ or depressi$ or bipolar or unipolar or schizophren$ or epilep$ or seizure or dementia or Parkinson$ or anorex$ or bulim$ or obsessive or compulsive or anxiety or multiple sclerosis or insomni$ or migrain$ or headache or head ache or addict$ or withdraw$ or abstinen$ or non-communicable or noncommunicable or NCD$ [Words] |
| 4 | 2 or 3 |
| **5** | **1 and 4** |

Database: IMSEAR

| **#** | **Search term** |
| --- | --- |
| 1 | heart OR vascular OR blood OR cardiovascular OR cerebrovascular OR coronary OR atrial OR ventricular OR fibrillat* OR myocard* OR hypertensi* OR stroke OR angina OR aneurysm OR asthm* OR COPD OR chronic obstructive OR arthrit* OR gout* OR rheumat* OR diabet* OR depressi* OR bipolar OR unipolar OR schizophren* OR epilep* OR seizure OR dementia OR Parkinson* OR anorex* OR bulim* OR obsessive OR compulsive OR anxiety OR multiple sclerosis OR insomni* OR migrain* OR headache OR head ache OR addict* OR withdraw* OR abstinen* OR non-communicable OR noncommunicable OR NCD* |
| 2 | earning* OR income OR poverty OR expense* OR out-of-pocket OR OOP OR payment* OR expenditure* OR labor OR labour OR workforce OR productiv* OR absenteeism OR employment OR employab* OR gross domestic product OR national income OR economic growth OR cost of illness OR cost-of-illness OR burden of illness OR burden-of-illness OR economic burden OR economic consequences OR economic model |
| **3** | **1 and 2** |

Database: IMEMR

| **#** | **Search term** |
| --- | --- |
| 1 | "CARDIOVASCULAR DISEASES/" or "ASTHMA" or "CHRONIC OBSTRUCTIVE PULMONARY DISEASE/" or "NEOPLASM" or "DIABETES" or "MUSCULOSKELETAL DISEASES/" or "MENTAL DISORDERS" or "MENTAL HEALTH" or "NEUROLOGY/" or "NEUROMUSCULAR DISEASES/" or "BACK PAIN/" or "NECK PAIN/" [Words] |
| 2 | heart or vascular or blood or cardiovascular or cerebrovascular or coronary or atrial or ventricular or fibrillat$ or myocard$ or hypertensi$ or stroke or angina or aneurysm or asthm$ or COPD or chronic obstructive or arthrit$ or gout$ or rheumat$ or diabet$ or depressi$ or bipolar or unipolar or schizophren$ or epilep$ or seizure or dementia or Parkinson$ or anorex$ or bulim$ or obsessive or compulsive or anxiety or multiple sclerosis or insomni$ or migrain$ or headache or head ache or addict$ or withdraw$ or abstinen$ or non-communicable or noncommunicable or NCD$ |
| 3 | 1 or 2 |
| 4 | earning$ or income or poverty or expense$ or out-of-pocket or OOP or payment$ or expenditure$ or labor or labour or workforce or productiv$ or absenteeism or employment or employab$ or gross domestic product or national income or economic growth or cost of illness or cost-of-illness or burden of illness or burden-of-illness or economic burden or economic consequences or economic model [Words] |
| **5** | **3 and 4** |

Database: WPRIM

| **#** | **Search term** |
| --- | --- |
| 1 | "CARDIOVASCULAR DISEASES/" or "ASTHMA" or "CHRONIC OBSTRUCTIVE PULMONARY DISEASE/" or "NEOPLASM" or "DIABETES" or "MUSCULOSKELETAL DISEASES/" or "MENTAL DISORDERS" or "MENTAL HEALTH" or "NEUROLOGY/" or "NEUROMUSCULAR DISEASES/" or "BACK PAIN/" or "NECK PAIN/" |
| 2 | heart or vascular or blood or cardiovascular or cerebrovascular or coronary or atrial or ventricular or fibrillat$ or myocard$ or hypertensi$ or stroke or angina or aneurysm or asthm$ or COPD or chronic obstructive or arthrit$ or gout$ or rheumat$ or diabet$ or depressi$ or bipolar or unipolar or schizophren$ or epilep$ or seizure or dementia or Parkinson$ or anorex$ or bulim$ or obsessive or compulsive or anxiety or multiple sclerosis or insomni$ or migrain$ or headache or head ache or addict$ or withdraw$ or abstinen$ or non-communicable or noncommunicable or NCD$ |
| 3 | 1 or 2 |
| 4 | earning$ or income or poverty or expense$ or out-of-pocket or OOP or payment$ or expenditure$ or labor or labour or workforce or productiv$ or absenteeism or employment or employab$ or gross domestic product or national income or economic growth or cost of illness or cost-of-illness or burden of illness or burden-of-illness or economic burden or economic consequences or economic model |
| **5** | **3 and 4** |
