# Supplementary material for: The economic burden of cardiovascular disease and hypertension in low- and middle-income countries: a systematic review
Source: BMC Public Health. 2018 Aug 6;18:975. doi: 10.1186/s12889-018-5806-x (PMC6090747; doi:10.1186/s12889-018-5806-x)
Supplement: Supplementary file 2 — Quality assessment tool. (DOCX 14 kb) [file 12889_2018_5806_MOESM2_ESM.docx]

Additional file 2 Quality assessment tool

Economic component [Yes/No/Unclear]

Q1. Were data sources for expenditure, resource use and unit costs clearly explained?

Q2. Were cost and/or expenditure data transparently presented?

Q3. Have productivity costs been included?

Q4. If productivity costs were included, were results presented with and without these?

Q5. Did the analysis address uncertainty and/or heterogeneity? E.g. through sensitivity analysis or subgroup analysis

Epidemiologic component (if applicable) [Yes/No/Unclear]

Q1. Is the patient sampling method appropriate for deriving nationwide estimates of incidence/prevalence?

Q2. Does the source of incidence/prevalence data contribute to the study’s internal validity?
