# Supplementary material for: The economic burden of cardiovascular disease and hypertension in low- and middle-income countries: a systematic review
Source: BMC Public Health. 2018 Aug 6;18:975. doi: 10.1186/s12889-018-5806-x (PMC6090747; doi:10.1186/s12889-018-5806-x)
Supplement: Supplementary file 3 — Selected descriptive characteristics of included studies. (DOCX 208 kb) [file 12889_2018_5806_MOESM3_ESM.docx]

Additional file 3 Selected descriptive characteristics of included studies

| Study ID | Region/country | **Condition** | **Delivery setting** | **Economic perspective** | **Study design** | **Type of economic estimate(s)** |
| --- | --- | --- | --- | --- | --- | --- |
|  |  |  |  |  |  |  |
|  |  |  |  |  |  |  |
| Adoukonou, 2013 #3842[1] | Benin | stroke | tertiary care | societal | prospective cost study | total cost of stroke episode |
| Akanksha, 2013 #77[2] | India | all cardiovascular diseases | secondary care | provider | mathematical model | mean cost/hospitalization for CVD. total cost of hospitalization for CVDs |
| Alam, 2014 #2145[3] | Bangladesh, India, Nepal and Sri Lanka | angina | other | patient | database analysis | OOP spending for hospitalization, drugs and medicines; borrowing or selling assets to afford health expenditure; %OOP spending of household expenditure |
| Alefan, 2009 #23988[4] | Malaysia | hypertension | other | other | retrospective cost study | cost/month |
| Al-Junid, 2007 #46665[5] | Malaysia | any cardiovascular disease as recorded using IR DRG | tertiary care | provider | retrospective cost study | drug cost/patient episode |
| Altagracia-Martinez, 2006 #47414[6] | Mexico | hypertension | other | other | database analysis | cardiovascular agents marker |
| Aounallah Skhiri, 2005 #10882[7] | Tunisia | myocardial infarction | secondary care | other | prospective cost study | average cost per stay |
| Araujo, 2005 #48811[8] | Brazil | heart failure | other | societal | retrospective cost study | average cost per patient |
| Araujo, 2013 #13106[9] | Brazil | myocardial infarction | secondary care | payer | database analysis | average hospitalization cost |
| Arredondo, 1995 #5799[10] | Brazil | cardiac valve disease; breast cancer | secondary care | provider | retrospective cost study | cost/case |
| Arredondo, 1997 #34745[11] | Mexico | hypertension; diabetes | other | provider | retrospective cost study | cost/case; cost/state |
| Arredondo, 2006 #4644[12] | Mexico | hypertension | other | payer | CoI study | cost/country |
| Asil, 2011 #19814[13] | Turkey | stroke | secondary care | provider | retrospective cost study | cost/case |
| Azambuja, 2008 #61692[14] | Brazil | severe cardiovascular disease | other | societal | COI study | total national cost; total national cost as % GDP; total cost/case |
| Birabi, 2012 #7110[15] | Nigeria | stroke | other | provider | retrospective cost study | average cost/case |
| Bloom, 2013 #93[16] | China, India | cardiovascular disease | other | societal | mathematical model | economic loss due to disease |
| Bovet, 2006 #4662[17] | Seychelles | hypertension | primary care | provider | mathematical model | average annual cost for hypertension management |
| Calvo-Vargas, 1998 #28035[18] | Mexico | hypertension | primary care | provider | mathematical model | annual cost per patient of hypertension treatment |
| Castaneda-Cardona, 2014 #22088[19] | Colombia | ischemic stroke | tertiary care | unclear | retrospective cost study | total cost of stroke episode |
| Chen, 2003 #31495[20] | China | hypertension | primary care | payer | database analysis | median yearly cost per patient of outpatient care |
| Christensen, 2009 #24586[21] | Argentina | intracerebral hemorrhage and ischemic stroke | secondary care | provider | other | average cost/case; national cost |
| Christensen, 2009 #24766[22] | Brazil | stroke | secondary care | provider | other | average cost/case; national cost |
| Cook, 2014[23] | Global | heart failure | other | societal | mathematical model | direct costs per annum; indirect costs per annum; %GDP |
| Diaz, 2006 #47318[24] | Chile | stroke | tertiary care | provider | retrospective cost study | average cost per case |
| Dib, 2010 #2943[25] | Brazil | hypertension | other | provider | mathematical model | total national direct cost; direct cost % GDP |
| Dorohoi, 2004 #30698[26] | Ukraine | multiple cardiovascular | primary care | provider | retrospective cost study | average annual cost per patient |
| Engelgau, 2012 #1230[27] | India | heart disease; hypertension | other | patient | COI study | average OOP cost per patient per hospital stay |
| Fernandez Garcia, 2008 #3500[28] | Cuba | myocardial infarction | tertiary care | provider | retrospective cost study | average cost per case |
| Finkelstein 2014[29] | Indonesia | hypertension | other | patient | survey | average OOP annual burden per case |
| Fong Estrada, 2011 #60903[30] | Cuba | hypertension | secondary care | provider | retrospective cost study | average cost per case |
| Gao, 2005 #30377[31] | China | stroke | tertiary care | provider | retrospective cost study | average cost per case |
| Gaziano, 2009 #42320[32] | Sub-Saharan Africa | nonoptimal blood pressure | other | payer | mathematical model | annual healthcare cost of nonoptimal blood pressure; annual healthcare cost of MI/stroke; % healthcare expenditure attributed to high blood pressure; welfare loss attributable to non-optimal blood pressure; %GDP lost from non-optimal blood pressure |
| Gombet, 2009 #23574[33] | Congo | stroke; heart failure; hypertension | secondary care | provider | retrospective cost study | average cost per hospitalization |
| Heeley, 2009 #23695[34] | China | stroke | other | other | prospective cost study | total medical expenses in first 3 months; OOP expenses in first 3 months; OOP expenses as % of total annual household income; % patients experiencing catastrophic payments |
| Hejazi, 2015 #13341[35] | Malaysia | stroke | tertiary care | patient | prospective cost study | total cost of stroke care |
| Hu, 2013 #13652[36] | China | atrial fibrillation-related stroke in China | other | societal | retrospective cost study | direct costs during acute hospitalization; direct costs 1 year after discharge; indirect costs |
| Huffman, 2011 #39365[37] | Argentina, India, China, Tanzania | acute coronary syndrome (including unstable angina or MI), stroke, acute heart failure, peripheral vascular intervention | other | patient | retrospective cost study | OOP; catastrophic health spending; distress financing; decreased work time. |
| Ilesanmi, 2012 #916[38] | Nigeria | primary hypertension | other | patient | retrospective cost study | OOP |
| Ivanova, 2009 #24356[39] | Bulgaria, Serbia | hypertension | other | other | database analysis | prescription cost; payer's participation in prescription cost |
| Kabadi, 2013 #9261[40] | Tanzania | stroke | other | societal | prospective cost study | average total cost at 6-months post-stroke |
| Karan, 2014 #3377[41] | India | cardiovascular disease | other | patient | database analysis | OOP spending for hospitalization, drugs, outpatient visits, transport, non-medical spending |
| Kauf, 2006 #48176[42] | Argentina | acute myocardial infarction | secondary care | other | database analysis | hospitalization cost |
| Khealani, 2003 #5154[43] | Pakistan | acute stroke | tertiary care | provider | retrospective cost study | hospitalization cost |
| Khiaocharoen, 2012 #37052[44] | Thailand | acute and sub-acute stroke | secondary care | provider | prospective cost study | hospitalization cost |
| Kiatchoosakun, 2012 #120[45] | Thailand | coronary heart disease: angina, acute myocardial infarction and chronic ischemic heart disease | secondary care | payer | database analysis | hospital charges |
| Konin, 2007 #27560[46] | Cote d'Ivoire | acute myocardial infarction | secondary care | other | retrospective cost study | total cost |
| Kontsevaya 2013[47] | Russian Federation | cardiovascular diseases | other | societal | COI study | total direct and indirect cost; %GDP |
| Lakic, 2014 #11781[48] | Serbia | cardiovascular disease: hypertension, CHD, cardiompyopathy, heart failure, cerebrovascular disease | other | societal | COI study | total annual cost of CVD |
| Larijani, 2003#SP129036[49] | Iran | coronary heart disease | secondary care | other | retrospective cost study | hospitalization cost; number of days off work |
| Le, 2012 #703[50] | China | hypertension | other | societal | COI study | cost (time frame unclear) |
| Le, 2015 #2314[51] | China | coronary heart disease | other | societal | COI study | average annual cost |
| Long, 2005 #48354[52] | China | stroke | other | patient | retrospective cost study | medial annual direct cost per patient of stroke care |
| Long, 2007 #27501[53] | China | stroke | other | societal | other | indirect economic burden of stroke |
| Lucia Rios Nunez, 2008 #26204[54] | Mexico | hypertension | tertiary care | provider | retrospective cost study | average cost per hospitalization |
| Ma, 2010 #20469[55] | China | stroke | tertiary care | provider | retrospective cost study | cost |
| Mahal, 2010 #SP[56] | India | all cardiovascular diseases | other | societal | COI study | total national cost; income loss |
| Maharaj, 2012 #11744[57] | Fiji Islands | stroke | other | societal | other | annual national human capital loss |
| Malhotra, 2000 #6091[58] | India | angina | tertiary care | patient | prospective cost study | average cost per patient of angina hospitalization |
| Marfatia, 2014 #9326[59] | India | stroke related to nonvalvular atrial fibrillation | tertiary care | societal | retrospective cost study | average annual cost of stroke care |
| Marques, 2012 #16320[60] | Brazil | myocardial infarction | tertiary care | provider | retrospective cost study | average cost per hospitalization |
| Marteau, 2001 #33411[61] | Argentina | hypertension, dyslipidemia and diabetes | primary care | provider | retrospective cost study | average cost per consultation |
| Meena, 2012 #644[62] | India | acute coronary syndrome | other | patient | retrospective cost study | out of pocket expenditure 3 months pre-event and 6 months post-event (9months total); catastrophic spending |
| Mohd Nordin, 2012 #15224[63] | Malaysia | stroke | tertiary care | provider | retrospective cost study | average cost of care |
| Moleerergpoom, 2007 #27061[64] | Thailand | coronary acute syndrome | secondary care | patient | database analysis | cost of hospitalization |
| Murphy, 2013 #131[65] | Ukraine | angina | other | patient | database analysis | per capita monthly household expenditure |
| Ogah, 2014 #13682[66] | Nigeria | heart failure | tertiary care | societal | retrospective cost study | average annual cost of HF management |
| Pestana, 1996 #5806[67] | South Africa | generic cardiovascular | other | societal | COI study | total national cost |
| Reynales-Shigematsu, 2006 #4585[68] | Mexico | acute myocardial infarction attributable to tobacco consumption | other | provider | retrospective cost study | annual average cost per case; total annual costs attributable to tobacco consumption |
| Ribeiro, 2005 #30289[69] | Brazil | coronary heart disease | other | provider | prospective cost study | average annual cost per patient |
| Rubinstein, 2010 #12814[70] | Argentina | acute coronary heart disease and stroke | secondary care | other | mathematical model | national annual cost of CHD and stroke care |
| Sadoh, 2011 #10885[71] | Nigeria | chronic heart failure in children with congenital heart disease | secondary care | patient | prospective cost study | total monthly expenditure per family |
| Shu, 2010 #2492[72] | China | stroke | other | patient | COI study | average economic burden of stroke per patient |
| Sozmen, 2015 #1214[73] | Turkey | angina, myocardial infarction, heart failure | tertiary care | payer | retrospective cost study | average direct cost per admission |
| Suhil, 2010 #21952[74] | Malaysia | hypertension | secondary care | provider | prospective cost study | total cost per patient per year |
| Toure, 2005 #48281[75] | Senegal | stroke | tertiary care | provider | prospective cost study | average cost per hospitalization |
| Tu, 2002 #32915[76] | China | stroke | secondary care | patient | retrospective cost study | total charges per inpatient stay |
| Villarreal-Rios, 2002 #5431[77] | Mexico | hypertension | primary care | other | retrospective cost study | average annual cost per patient |
| Wei, 2010 #40488[78] | China | stroke | secondary care | patient | COI study | total charges per inpatient stay |
| World Bank, 2005 #SP[79] | Russian Federation | generic cardiovascular disease | other | societal | COI study | total national cost of CVD |
| Yang, 2008 #26380[80] | China | generic cardiovascular disease | other | patient | COI study | average annual cost of CVD care |
| Zambrana, 2008 #3823[81] | Mexico | hypertension | secondary care | payer | database analysis | total national cost of CVD |
| Zhai, 2006 #47308[82] | China | generic cardiovascular disease | other | provider | COI study | mean annual cost of primary prevention for stroke |
| Zhao, 2013 #4378[83] | China | high risk of ischemic stroke | secondary care | societal | retrospective cost study |  |

References

1. Adoukonou, T., et al., *[Direct hospital cost of stroke in Parakou in northern Benin].* The Pan African medical journal, 2013. **16**: p. 121.

2. Akanksha, S. and S.K. Mohanty, *Age and sex pattern of cardiovascular mortality, hospitalisation and associated cost in India.* PLoS ONE, 2013. **8**(5).

3. Alam, K. and A. Mahal, *The economic burden of angina on households in South Asia.* BMC Public Health, 2014. **14**: p. 179.

4. Alefan, Q., et al., *Cost of treating hypertension in Malaysia.* Asian Journal of Pharmaceutical and Clinical Research, 2009. **2**(1): p. 1-5.

5. Al-Junid, S.M., W.P. Ezat, and S. Surianti, *Prescribing patterns and drug cost among cardiovascular patients in Hospital Universiti Kebangsaan Malaysia.* Medical Journal of Malaysia, 2007. **62**(1): p. 59-65.

6. Altagracia-Martinez, M., et al., *Consumption and costs of antihypertensive drugs in Mexico: are diuretic agents a standing technological trajectory?* Research In Social & Administrative Pharmacy, 2006. **2**(1): p. 22-37.

7. Aounallah Skhiri, H., et al., *The cost of acute myocardial infarction management: the Tunisian experience.* Cout direct medical de la prise en charge de l'infarctus du myocarde en phase aigue, 2005. **83**(5): p. 24-29.

8. Araujo, D.V., et al., *[Cost of heart failure in the Unified Health System].* Arquivos Brasileiros de Cardiologia, 2005. **84**(5): p. 422-7.

9. Araujo, D.V., L. Bahia, and S.F. Stella, *The Economic Burden of HIV/AIDS and myocardial infarction treatment in Brazil.* Thescientificworldjournal, 2013. **2013**: p. 864962.

10. Arredondo, A., L.Y. Lockett, and E.d. Icaza, *Cost of diseases in Brazil: breast cancer, enteritis, cardiac valve disease and bronchopneumonia.* Revista de Saude Publica, 1995. **29**(5): p. 349-354.

11. Arredondo, A., *Costs and financial consequences of the changing epidemiologogical profile in Mexico.* Health Policy, 1997. **42**(1): p. 39-48.

12. Arredondo, A. and A. Zuniga, *Epidemiologic changes and economic burden of hypertension in Latin America: evidence from Mexico.* American Journal of Hypertension, 2006. **19**(6): p. 553-559.

13. Asil, T., et al., *Cost of acute ischemic and hemorrhagic stroke in Turkey.* Clinical Neurology and Neurosurgery, 2011. **113**(2): p. 111-114.

14. Azambuja, M.I.R., et al., *Economic burden of severe cardiovascular diseases in Brazil: An estimate based on secondary data. [Portuguese, English]*

*Impacto economico dos casos de doenca cardiovascular grave no Brasil: Uma estimativa baseada em dados secundarios.* Arquivos Brasileiros de Cardiologia, 2008. **91**(3): p. 148-155+163-171.

15. Birabi, B.N., et al., *Cost burden of post stroke condition in Nigeria: a pilot study.* Global journal of health science, 2012.

16. Bloom, D.E., et al. *The Economic Impact of Non-Communicable Disease in China and India: Estimates, Projections, and Comparisons*. 2013 2013//.

17. Bovet, P., et al., *Prevalence of cardiovascular risk factors in a middle-income country and estimated cost of a treatment strategy.* BMC Public Health, 2006. **6**(9).

18. Calvo-Vargas, C.G., et al., *Changes in the costs of antihypertensive medications in a developing country: a study in Mexico comparing 1990 and 1996.* American Journal of Hypertension, 1998. **11**(4 Pt 1): p. 487-93.

19. Castañeda-Cardona, C., et al., *Análisis de costos de atención de infarto cerebral agudo con o sin fibrilación auricular^ies*

*Cost analysis of acute stroke care with or without atrial fibrillation^ien.* Acta neurol. colomb, 2014. **30**(2): p. 78-82.

20. Chen, Y., et al., *An analysis on the cost of hypertensive outpatient in the community hospital in Shanghai. [Chinese].* Zhonghua liu xing bing xue za zhi = Zhonghua liuxingbingxue zazhi, 2003. **24**(12): p. 1074-1077.

21. Christensen, M.C., et al., *Acute treatment costs of intracerebral hemorrhage and ischemic stroke in Argentina.* Acta Neurologica Scandinavica, 2009. **119**(4): p. 246-253.

22. Christensen, M.C., et al., *Acute treatment costs of stroke in Brazil.* Neuroepidemiology, 2009. **32**(2): p. 142-149.

23. Cook, C., et al., *The annual global economic burden of heart failure.* International Journal of Cardiology, 2014. **171**(3): p. 368-76.

24. Diaz, T.V., et al., *[Evaluation of a stroke unit at a university hospital in Chile].* Revista Medica de Chile, 2006. **134**(11): p. 1402-8.

25. Dib, M.W., R. Riera, and M.B. Ferraz, *Estimated annual cost of arterial hypertension treatment in Brazil.* Revista Panamericana de Salud Publica/Pan American Journal of Public Health, 2010. **27**(2): p. 125-131.

26. Dorohoi, A.P., et al., *Estimated cost of ambulatory treatment of patients with cardiovascular diseases. [Ukrainian]*

*Oriientovna vartist' likuvannia khvorykh iz sertsevo-sudynnymy zakhvoriuvanniamy v ambulatornykh umovakh.* Likars'ka sprava / Ministerstvo okhorony zdorov'ia Ukrainy, 2004(5-6): p. 93-99.

27. Engelgau, M.M., A. Karan, and A. Mahal, *The economic impact of Non-Communicable Diseases on households in India.* Globalization and Health, 2012. **8**(9).

28. Fernandez Garcia, A., A.M. Galvez Gonzalez, and A. Castillo Guzman, *Institutional cost of acute myocardial infarction at the Institute of Cardiology and Cardiovascular Surgery.* Revista Cubana de Salud Publica, 2008. **34**(4).

29. Finkelstein, E.A., J. Chay, and S. Bajpai, *The economic burden of self-reported and undiagnosed cardiovascular diseases and diabetes on Indonesian households.* PLoS ONE [Electronic Resource], 2014. **9**(6): p. e99572.

30. Fong Estrada, J.A., E. Zayas Esteven, and J. Oliva Fong, *Costo del tratamiento antihipertensivo en ancianos ingresados en los servicios de Geriatría y Medicina Interna^ies*

*Cost of antihypertensive treatment in elderly admitted to the services of Geriatrics and Internal Medicine^ien.* Medisan, 2011. **15**(1): p. 67-72.

31. Gao, X.F., et al., *Comparative study of costs by case-mix model for stroke inpatients. [Chinese].* Chinese Journal of Evidence-Based Medicine, 2005. **5**(1): p. 42-46+85.

32. Gaziano, T.A., et al., *The global cost of nonoptimal blood pressure.* Journal of Hypertension, 2009. **27**(7): p. 1472-7.

33. Gombet, T.R., et al., *Cost of emergency cardiovascular care at the University Hospital Center in Brazzaville, Congo.* Med Trop, 2009. **69**.

34. Heeley, E., et al., *Role of health insurance in averting economic hardship in families after acute stroke in China.* Stroke, 2009. **40**.

35. Hejazi, S.M.A., et al., *Cost of post-stroke outpatient care in Malaysia.* Singapore Medical Journal, 2015. **56**(2): p. 116-119.

36. Hu, S., et al., *Economic burden of individual suffering from atrial fibrillation-related stroke in China.* Value in Health Regional Issues, 2013. **2**(1): p. 135-140.

37. Huffman, M.D., et al., *A Cross-Sectional Study of the Microeconomic Impact of Cardiovascular Disease Hospitalization in Four Low- and Middle-Income Countries.* PLoS ONE, 2011. **6**(6): p. e20821.

38. Ilesanmi, O.S., O.K. Ige, and A.O. Adebiyi, *The managed hypertensive: the costs of blood pressure control in a Nigerian town.* Pan African Medical Journal, 2012. **12**(17).

39. Ivanova, A., et al., *Cost of outpatient hypertension pharmacotherapy: Comparative study between Bulgaria and Serbia.* Pharmacy Practice, 2009. **7**(2): p. 108-112.

40. Kabadi, G.S., et al., *The cost of treating stroke in urban and rural Tanzania: A 6-month pilot study*

*Le cout du traitement des accidents vasculaires cerebraux en Tanzanie urbaine et rurale: Une etude pilote 6 MOIS.* African Journal of Neurological Sciences, 2013. **32**(2).

41. Karan, A., M. Engelgau, and A. Mahal, *The household-level economic burden of heart disease in India.* Tropical Medicine & International Health, 2014. **19**(5): p. 581-91.

42. Kauf, T.L., et al., *The cost of acute myocardial infarction in the new millennium: evidence from a multinational registry.* American Heart Journal, 2006. **151**(1): p. 206-12.

43. Khealani, B.A., et al., *Cost of acute stroke care at a tertiary care hospital in Karachi, Pakistan.* JPMA, Journal of the Pakistan Medical Association, 2003. **53**(11): p. 552-555.

44. Khiaocharoen, O., S. Pannarunothai, and C. Zungsontiporn, *Cost of acute and sub-acute care for stroke patients.* Journal of the Medical Association of Thailand, 2012. **95**(10): p. 1266-77.

45. Kiatchoosakun, S., S. Sutra, and K. Thepsuthammarat, *Coronary artery disease in the Thai population: data from health situation analysis 2010.* Journal of the Medical Association of Thailand, 2012. **95**(7 Supplement 7): p. 149-155.

46. Konin, C., et al., *Assessment of the cost of the curative treatment of myocardial infarction in the Abidjan Cardiology Institute. [French]*

*Evaluation du cout du traitement curatif de l'infarctus du myocarde a l'Institut de cardiologie d'Abidjan (ICA), Cote d'Ivoire.* Bulletin de la Societe de pathologie exotique (1990), 2007. **100**(3): p. 201-204.

47. Kontsevaya, A., A. Kalinina, and R. Oganov, *Economic burden of cardiovascular diseases in the Russian federation.* Value in Health Regional Issues, 2013. **2**(2): p. 199-204.

48. Lakic, D., L. Tasic, and M. Kos, *Economic burden of cardiovascular diseases in Serbia*

*Kardiovaskularne bolesti u Srbiji - Ekonomski teret.* Vojnosanitetski Pregled, 2014. **71**(2): p. 137-143.

49. Larijani, B., et al., *Burden of coronary heart disease on the Iranian oil industry (1999–2000).* La Revue de Sante de la Mediterranee orientale, 2003. **9**(5/6): p. 904-910.

50. Le, C., et al., *The economic burden of hypertension in rural south-west China.* Tropical Medicine and International Health, 2012. **17**(12): p. 1544-1551.

51. Le, C., et al., *Economic burden and cost determinants of coronary heart disease in rural southwest China: a multilevel analysis.* Public Health, 2015. **129**(1): p. 68-73.

52. Long, Y., et al., *[Analysis on direct economic burden of stroke in the rural population of Hanzhong, Shaanxi Province].* Chung-Hua Liu Hsing Ping Hsueh Tsa Chih Chinese Journal of Epidemiology, 2005. **26**(7): p. 494-7.

53. Long, Y., et al., *Evaluation on the indirect economic burden of stroke using combination of disability-adjusted life years and human capital method. [Chinese].* Zhonghua liu xing bing xue za zhi = Zhonghua liuxingbingxue zazhi, 2007. **28**(7): p. 708-711.

54. Lucia Rios Nunez, L.C., *Attention cost per hospitalization of patients with hypertensive diseases at the Ignacio Chavez National Institute of Cardiology. [Spanish]*

*Costo de atencion hospitalaria de pacientes con enfermedades hipertensivas en el Instituto Nacional de Cardiologia Ignacio Chavez.* Revista Mexicana de Enfermeria Cardiologica, 2008. **16**(3): p. 82-86.

55. Ma, Y., et al., *Evaluation of admission characteristics, hospital length of stay and costs for cerebral infarction in a medium-sized city in China.* European Journal of Neurology, 2010. **17**(10): p. 1270-1276.

56. Mahal, A., A. Karan, and M. Engelgau, *The Economic Implications of Non-Communicable Disease for India*. World Bank/HNP Discussion Paper. 2010, Washington, DC: World Bank.

57. Maharaj, J.C. and M. Reddy, *Young stroke mortality in fiji islands: an economic analysis of national human capital resource loss.* ISRN neurology, 2012.

58. Malhotra, S., et al., *A study of drug utilisation and cost of treatment in patients hospitalised with unstable angina.* European Journal of Clinical Pharmacology, 2000. **56**(9/10): p. 755-761.

59. Marfatia, S., et al., *Treatment costs of stroke related to nonvalvular atrial fibrillation patients in India-a multicenter observational study.* Value in Health Regional Issues, 2014. **3**(1): p. 205-210.

60. Marques, R., et al., *Costs of the chain of treatment procedures in acute myocardial infarction in Brazilian reference and specialized hospitals*

*Custos da cadeia de procedimentos no tratamento do infarto agudo do miocardio em hospitais brasileiros de excelencia e especializados.* Revista da Associacao Medica Brasileira, 2012. **58**(1): p. 104-111.

61. Marteau, S.A. and L.H. Perego, *Activity-based cost model applied to tracer cardiovascular diseases. [Spanish]*

*Modelo del costo basado en la actividad aplicado a consultas por trazadores de enfermedades cardiovasculares.* Salud Publica de Mexico, 2001. **43**(1): p. 32-40.

62. Meena, D., et al., *Catastrophic health expenditure & coping strategies associated with acute coronary syndrome in Kerala, India.* Indian Journal of Medical Research, 2012. **136**(4): p. 585-592.

63. Mohd Nordin, N.A., et al., *Direct medical cost of stroke: Findings from a tertiary hospital in Malaysia.* Medical Journal of Malaysia, 2012. **67**(5): p. 473-477.

64. Moleerergpoom, W., et al., *Costs of payment in Thai acute coronary syndrome patients.* Journal of the Medical Association of Thailand = Chotmaihet thangphaet, 2007. **90 Suppl 1**: p. 21-31.

65. Murphy, A., et al., *The economic burden of chronic disease care faced by households in Ukraine: a cross-sectional matching study of angina patients.* International Journal for Equity in Health, 2013. **12**(38).

66. Ogah, O.S., et al., *Economic burden of heart failure: investigating outpatient and inpatient costs in Abeokuta, southwest Nigeria.* PLoS ONE, 2014. **9**(11).

67. Pestana, J.A.X., et al., *The direct and indirect costs of cardiovascular disease in South Africa in 1991.* South African Medical Journal, 1996. **86**(6): p. 679-684.

68. Reynales-Shigematsu, L.M., et al., *Costs of medical care for acute myocardial infarction attributable to tobacco consumption.* Archives of Medical Research, 2006. **37**(7): p. 871-879.

69. Ribeiro, R.A., et al., *Annual cost of ischemic heart disease in Brazil. Public and private perspective. [Portuguese]*

*Custo anual do manejo da cardiopatia isquemica cronica no Brasil. Perspectiva publica e privada.* Arquivos Brasileiros de Cardiologia, 2005. **85**(1): p. 3-8.

70. Rubinstein, A., et al., *Estimation of the burden of cardiovascular disease attributable to modifiable risk factors and cost-effectiveness analysis of preventative interventions to reduce this burden in Argentina.* BMC public health, 2010. **10**: p. 627.

71. Sadoh, W.E., D.U. Nwaneri, and A.C. Owobu, *The cost of out-patient management of chronic heart failure in children with congenital heart disease.* Nigerian Journal of Clinical Practice, 2011.

72. Shu, Z., L. Cai, and M. Yang, *Analysis on economic burden of stroke in a rural area of Lijiang Yunnan Province.* Modern Preventive Medicine, 2010. **37**(13): p. 2449-2450.

73. Sozmen, K., et al. *Determinants of inpatient costs of angina pectoris, myocardial infarction, and heart failure in a university hospital setting in Turkey*. 2015 [cited 15 (Sozmen, Pekel) Ministry of Health of Turkey, Izmir, Turkey]; 4:[325-333]. Available from: <http://www.anakarder.com/sayilar/115/buyuk/325-333.pdf>

<http://ovidsp.ovid.com/ovidweb.cgi?T=JS&PAGE=reference&D=emed13&NEWS=N&AN=2015893067>.

74. Suhil, M.A., M.A.A. Hassali, and M.I.M. Ibrahim, *Evaluation of direct medical cost in treating hypertension in a Malaysian public university.* Asian Journal of Pharmaceutical and Clinical Research, 2010. **3**(3): p. 170-173.

75. Toure, K., et al., *[Evaluation of the cost of stroke management in Dakar, Senegal].* Medecine Tropicale, 2005. **65**(5): p. 458-64.

76. Tu, F., et al., *Analysis of hospital charges for cerebral infarction stroke inpatients in Beijing, People's Republic of China.* Health Policy, 2002. **59**(3): p. 243-256.

77. Villarreal-Rios, E., et al., *The medical care costs of hypertension and their impact on health care resources in Mexico.* Salud Publica de Mexico, 2002. **44**(1): p. 7-13.

78. Wei, J.W., et al., *Variations and Determinants of Hospital Costs for Acute Stroke in China.* PLoS ONE, 2010. **5**(9): p. e13041.

79. World Bank, *Dying too Young: Addressing Premature Mortality and Ill Health Due to Non-Communicable Diseases and Injuries in the Russian Federation*. 2005, Washington, DC: World Bank.

80. Yang, L., et al., *Economic burden of cardiovascular diseases in China.* Expert Review of Pharmacoeconomics and Outcomes Research, 2008. **8**(4): p. 349-356.

81. Zambrana, M., et al., *Hospital spend for five pathologies of high economic impact.* Revista Medica del Instituto Mexicano del Seguro Social, 2008. **46**(1): p. 43-50.

82. Zhai, Y., et al., *[Economic burden of coronary heart disease and stroke attributable to hypertension in China].* Chung-Hua Liu Hsing Ping Hsueh Tsa Chih Chinese Journal of Epidemiology, 2006. **27**(9): p. 744-7.

83. Zhao, J.J., et al., *Status and costs of primary prevention for ischemic stroke in China.* Journal of Clinical Neuroscience, 2013. **20**(10): p. 1427-32.
