# Supplementary figures and images for: The economic burden of cardiovascular disease and hypertension in low- and middle-income countries: a systematic review
Source: BMC Public Health. 2018 Aug 6;18:975. doi: 10.1186/s12889-018-5806-x (PMC6090747; doi:10.1186/s12889-018-5806-x)

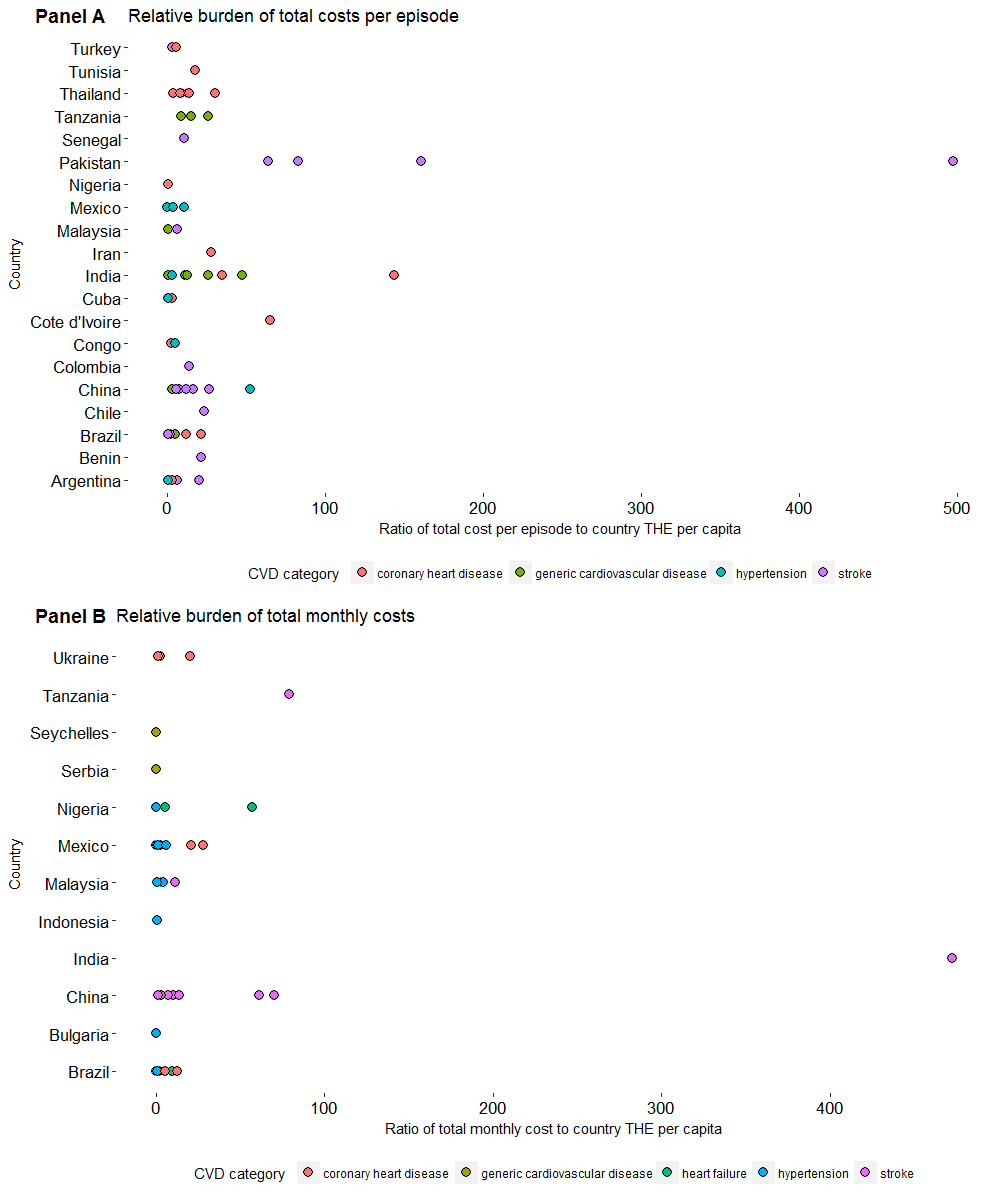

Supplement: Supplementary file 5 — Cost burden relative to total health expenditure per capita. (TIFF 3515 kb) [file 12889_2018_5806_MOESM5_ESM.tiff]
